# Dental and Mandibular Morphologies of *Arboroharamiya* (Haramiyida, Mammalia): A Comparison with Other Haramiyidans and *Megaconus* and Implications for Mammalian Evolution

**DOI:** 10.1371/journal.pone.0113847

**Published:** 2014-12-10

**Authors:** Jin Meng, Shundong Bi, Yuanqing Wang, Xiaoting Zheng, Xiaoli Wang

**Affiliations:** 1 Division of Paleontology, American Museum of Natural History, New York City, New York, United States of America; 2 Key Laboratory of Vertebrate Evolution and Human Origin of Chinese Academy of Sciences, Institute of Vertebrate Paleontology and Paleoanthropology, Chinese Academy of Sciences, Beijing, China; 3 Department of Biology, Indiana University of Pennsylvania, Indiana, Pennsylvania, United States of America; 4 Shandong Tianyu Museum of Nature, Pingyi, Shandong, China; 5 Institute of Geology and Paleontology, Linyi University, Linyi, Shandong, China; Monash University, Australia

## Abstract

**Background:**

Two recent studies published in the same issue of Nature reached conflicting conclusions regarding the phylogeny of early mammals: One places the clade containing haramiyidans and multituberculates within the Mammalia and the other separates haramiyidans from multituberculates and places the former outside of the Mammalia. These two contrasting results require that the minimally oldest divergence time of the Mammalia was within the Late Triassic or the Middle Jurassic, respectively. Morphological descriptions of the species named in the two papers were brief, and no comparisons between the newly named species were possible.

**Principal Findings:**

Here we present a detailed description of the dentary bone, teeth, occlusal and wear patterns of the haramiyidan *Arboroharamiya* and compare it with other haramiyidans and *Megaconus*. Using this new information, we suggest that tooth identifications and orientations of several previously described haramiyidan species are incorrect, and that previous interpretations of haramiyidan occlusal pattern are problematic. We propose that the published upper tooth orientation of *Megaconus* was problematic and question the number of upper molars, the length of dentition and mandible, and presence of the mandibular middle ear in *Megaconus*.

**Conclusions:**

The additional morphological descriptions and comparisons presented here further support the view that *Arboroharamiya*, as a derived haramiyidan, shows similarity to multituberculates in tooth and mandible morphologies. Our comparison also suggests that *Megaconus* lacks many diagnostic features for the family Eleutherodontidae and that its close affinity with multituberculates cannot be ruled out. The detailed morphological data demonstrate that haramiyidans are more similar to multituberculates than to any other mammaliaforms.

## Introduction

A major issue of mammalian evolution concerns the phylogenetic placement of Allotheria [1], including Haramiyida [2] and Multituberculata [3]. Multituberculates are the most diverse and best known group of Mesozoic mammals, morphologically resembling rodents [4], whereas haramiyidans remain poorly known, mainly from isolated teeth [5]–[15]. The oldest known multituberculates (*Kermackodon* and *Hahnotherium*) are from the Middle Jurassic (Late Bathonian) of Britain [13], [16]. The oldest haramiyidans, *Thomasia* and *Haramiyavia*, are from several localities of the Upper Triassic [20]. It has been postulated that the early occurrence of haramiyidans indicates an early diversification of allotherians from other mammals [11], [13], [17]–[20]. Allotherians were considered “so radically distinctive throughout their history that it seems hardly possible that they are related to other mammals except by a common origin at, or even before, the appearance of the class as such” ([17]: 168). This view is still held by some [13], [19], [21], whereas others place allotherians within the mammals [4], [22], [23] or within Theriiformes [24]. A common view of these studies and others [20] is that, if multituberculates and haramiyidans form the clade Allotheria, within Allotheria multituberculates form a monophyletic group that was derived from haramiyidans; thus, the latter form a paraphyletic group [13], [19], [21]. An alternative hypothesis is that multituberculates and haramiyidans are not related [25].

As the most diverse and best known group of Mesozoic mammals, multituberculates are represented by numerous craniodental and postcranial specimens [4]. In contrast, haramiyidans have remained enigmatic because all species of the group are known from isolated teeth except for *Haramiyavia clemmenseni* [25], which is based on dentitions and some cranial and postcranial remains. Because *Haramiyavia* appears morphologically primitive in having a postdentary trough (but see [15] and discussion) and other dental features, such as having the lower canine and four upper and lower incisors, the morphological gap remains large between it and other haramiyidans and multituberculates.

Recently, two Jurassic specimens from China have been reported as haramiyidans, both represented by dental and skeletal remains. In one study, *Arboroharamiya jenkinsi* was named and haramiyidans were clustered with multituberculates [26] to form a monophyletic allotherian clade placed within the Mammalia [27]. In another study, *Megaconus mammaliaformis* was named and haramiyidans were separated from multituberculates and placed outside of the Mammalia, as implied by the species name [28]. The phylogenetic discrepancy between the two studies implies different divergence times for the Mammalia, either within the Late Triassic [26] or within the Middle Jurassic [28].

While all phylogenetic hypotheses are subject to future testing and it will take time to resolve the discrepancy between competing phylogenetic hypotheses about haramiyidans and multituberculates, it is helpful to present more morphological data of the newly named species to the science community, particularly when some of the morphologies were considered to have not been clearly presented [29]. Therefore, with the introduction of the general morphologies of *Arboroharamiya* [26], here we follow up with a detailed description of the mandible and dentition of *Arboroharamiya*, accompanied by a discussion of issues raised by Zhou et al. [28] and Zheng et al. [26]. These issues include tooth identification in haramiyidans, tooth orientation and occlusion of haramiyidans, a comparison of *Arboroharamiya* with other haramiyidans and *Megaconus,* interpretation of the dentition of *Megaconus* (orientation, number of molars and dentition size), and the morphology of the middle ear of haramiyidans.

## Methods and Materials

The holotype specimen of *Arboroharamiya jenkinsi* (STM33-9, Tianyu Museum of Nature, Shandong Province, China) is preserved as two parts of a split slab, A and B, with slab A containing most of the skeletal elements [26]. The skull is not preserved except for fragments of the maxilla associated with the upper teeth. The two dentaries are preserved nearly intact. The loose teeth are preserved in isolation. Preparation has removed seven teeth from slab A so that their entirety can be observed. The teeth described here include both lower incisors, the left I1, left P3, both P4 s, right M1, right M2 (buccal half), left M2 (lingual half), both p4 s, both m1s and right m2.

We consider the sole upper incisor of *Arboroharamiya* as I1, the lower premolar as p4 and the two upper premolars as P3 and P4, respectively. We also consider the upper and lower molars as M1-2 and m1-2, respectively. These designations are for convenience of description; we do not assume any homology of these teeth with those having the same designations in other mammals. For instance, what is denoted as I1, may be actually homologous with I2 in multituberculates. This could be tested when better specimens of haramiyidans become available. However, we are aware that *Haramiyavia* was known to have four lower premolars and three upper and lower molars. If dental characters are coded in a phylogenetic analysis including haramiyidans, it seems most appropriate to compare the lower premolar of *Arboroharamiya* with p4 instead of other lower premolars of *Haramiyavia*. Similarly, the two upper and lower molars of *Arboroharamiya* are better compared with M1-2/m1-2 of *Haramiyavia*, respectively.

The optical photographs were taken using a Canon camera with a micro lens and the SEM photographs were taken using the Hitachi S-3700N scanning electron microscope. The measurements of teeth, already presented in Zheng et al. ([26], Suppl. Info.), were taken using a Mitutoyo digital caliper.

**Figure 1:**
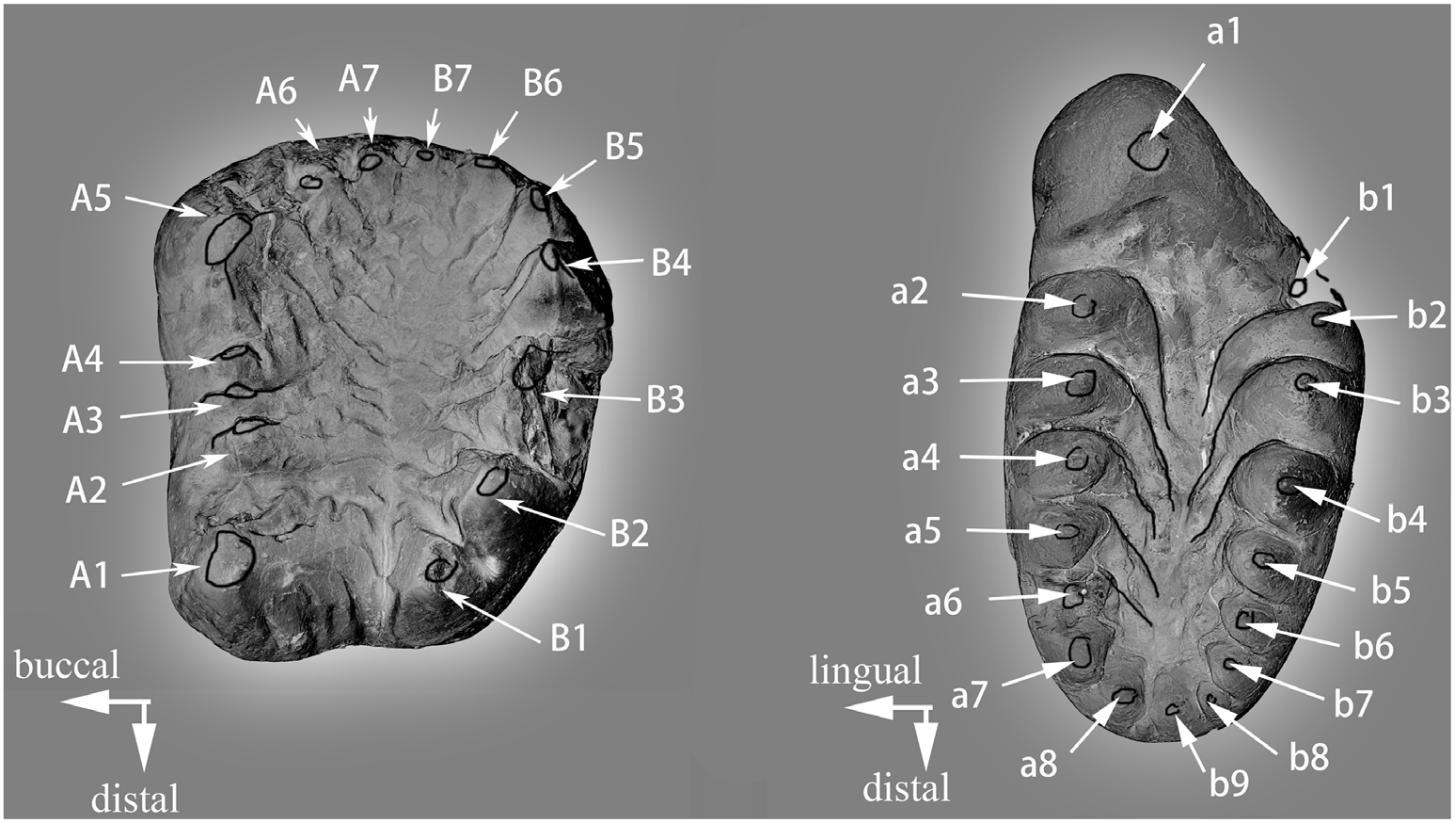
Dental orientation and terminology of *Arboroharamiya*. A. Cusp numbering in a right upper molar. B. Cusp numbering in a right lower molar. The terminology follows Butler [19].

For the dental descriptions we follow Butler [19] for the terminology of tooth cusps of haramiyidans, which is a nomenclatorial system developed from several studies [7], [10], [11], [25]. On upper molars the two rows of cusps are termed row A (buccal) and row B (lingual) with the cusps numbered from distal to mesial (Fig. 1A). For convenience of description we also number the small cusps mesial to A1, usually not numbered in eleutherodontids [13], [19], with the same numbering sequence. The two rows of cusps on the lower molar are termed row a (lingual) and row b (buccal) and the cusps are numbered from mesial to distal (Fig. 1B). Tooth replacement in haramiyidans is unknown, and for convenience of description we use premolar and molar instead of premolariform and molariform.

In *Haramiyavia* cusp b2 is the tallest in row b (buccal) [25]. Butler [19] attempted to homologize these cusps with those on the lower molar of *Thomasia.* He thought that cusp ‘b’ of *Thomasia* might be the homologue of bl of *Haramiyavia,* in which case cusp ‘bl’ of *Thomasia* would be homologous with b2 of *Haramiyavia.* We find it is difficult to make any homology statement about these cusps in *Arboroharamiya*; thus, for convenience of description, we number the tooth cusps purely by their position.

## Comparative Description

### Mandible

**Figure 2:**
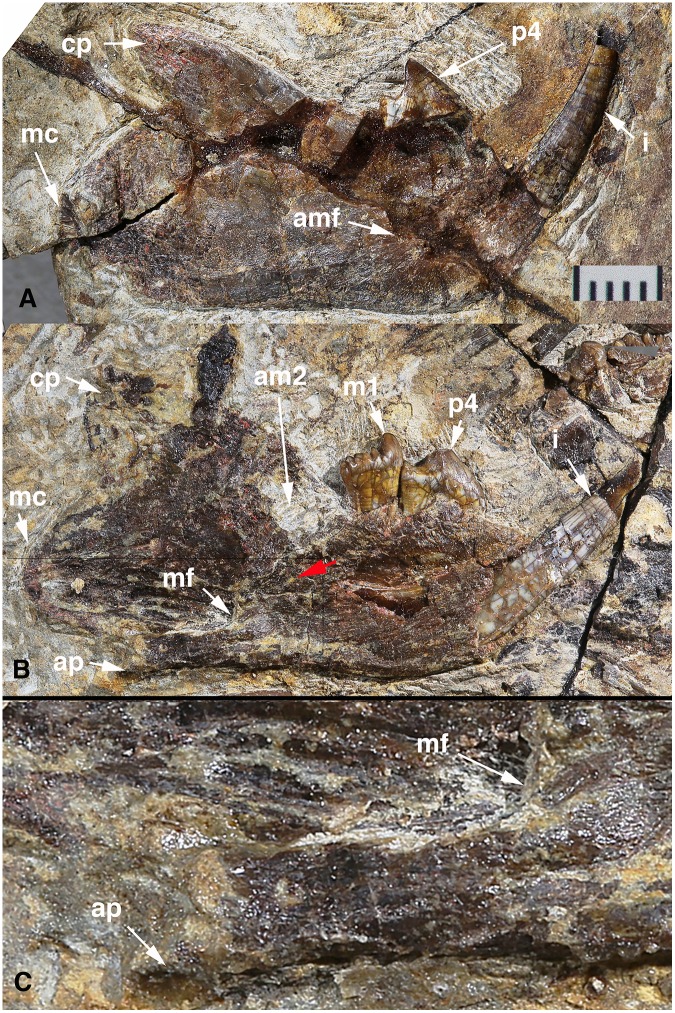
Mandibles of *Arboroharamiya jenkinsi.* A, Buccal view of the right mandible with p4 and the incisor (i). B, Lingual view of the left mandible with p4-m1 and the incisor. C, Close-up view of the angular process area of B, showing no groove or attachment site for the Meckel’s cartilage or the postdentary bones. Scale bar in A is 5 mm. Red arrow points to the possible coronoid bone. A and B are modified from Zheng et al. [26] A new arboreal haramiyid shows the diversity of crown mammals in the Jurassic period. Nature 500: 199–202 (DOI: 10.1038/nature12353). Reproduced by permission of Nature Publishing Group. Abbreviations: **ap**, angular process; **am2**, alveolus for m2; **amf**, anterior extremity of the masseteric fossa; **cp**, coronoid process; **mc**, mandibular condyle; **mf**, mandibular foramen.

Both dentaries are preserved. The buccal side of the right dentary and the lingual side of the left dentary are exposed (Fig. 2A–B). Only the incisor and p4 are preserved in situ in the right dentary, whereas the incisor, p4 and m1 are preserved in the left dentary. The alveolus for the left m2 is posterior to m1 and filled with matrix. The mandible is proportionally short and deep. The left dentary measures 31 mm (30 mm for the right one) from the anterior edge of the incisor alveolus to the posterior border of the mandibular condyle and 37.65 mm from the tip of the incisor to the posterior border of the condyle. The dentary depth on the medial side of the left mandible is 9.93 mm, measured from the alveolus edge of m1 to the bottom of the dentary; the depth on the lateral side of the right mandible is 11.65 mm, measured at the same position. The diastema between the incisor and p4 is distinct and about two-thirds of the length of p4. The diastema is short for a dentition that lacks a canine and has only one premolar.

The body of the dentary is dorsoventrally deep relative to the length, with its ventral edge being slightly concave below m2. The condyle is similar to that of multituberculates; it extends dorsoventrally instead of mediolaterally. The condyle has a semi-circular outline in lateral view and is situated lower than the tooth row. The coronoid process is small and triangular in lateral view, pointing posterodorsally. The dorsal border of the process thickens to form a rounded coronoid ridge that gradually narrows toward the tip of the process. The coronoid ridge and the blunt ridge along the ventral border of the dentary confine a broad but shallow masseteric fossa that ends anteriorly at the level of p4. The right dentary is broken at its anterior part on the lateral side, which may slightly alter the position of the incisor, the premolar and the coronoid process, but it is clear from the preserved part that the anteroventral border of the dentary is smoothly curved, as in multituberculates. There is no foramen on the preserved lateral surface of the mandible.

The left dentary shows that the symphysis is small, which indicates an unfused and movable symphysis for such a robust mandible. Posterior to the symphysis and anterior to the mandibular foramen, the medial surface of the dentary is smooth. Breakage on the medial side of the dentary reveals that the incisor extends posteriorly to the level of m2. The mandibular foramen is large and opens posteriorly into the pterygoid fossa. The pterygoid fossa is gently concave and extends from the mandibular foramen to the medial side of the condyle; the fossa is deeper anteriorly than posteriorly. Posteroventral to the m2 alveolus there seems to be a rudimentary coronoid bone that overlaps on the dentary and has an irregular suture.

Ventral to the pterygoid fossa is a blunt ridge with a smooth surface that terminates posteriorly as the angular process. Unlike multituberculates in which the ventral margin of the dentary is inflected and forms the pterygoideus shelf [4], the angular process of *Arboroharamiya* bends medially. It is clear that there is no groove in this area for the Meckel’s cartilage or any postdentary elements. Fig. 2C shows a magnified image of the region, since the original images and report for lack of the groove [26] were considered insufficient to determine the morphology [29]. Lack of the groove indicates detachment of the postdentary bones and thus acquisition of the middle ear ossicles.

### Lower Incisor

There is only one pair of enlarged lower incisors, considered as i1. The robust, presumably rootless, tooth gradually tapers toward its tip. The crown is completely covered with thin enamel, but it is difficult to determine how far posteriorly the enamel extends along the tooth within the jawbone. From the breakage of the jawbone, it can be seen that the incisor extends posteriorly below m2. The incisor crown is conical with an oval cross-section, contrasting with those that have a flat surface on the lingual (dorsal) side as seen in other haramiyidans and some multituberculates [20]. The incisor is proportionally longer but appears less procumbent (more vertical) than those of other haramiyidans and Jurassic multituberculates [4], [10], [20], [25], but this condition may be due to postmortem alteration.

*Arboroharamiya* does not have a canine, which differs from *Haramiyavia,* but is similar to *Megaconus* [28] and multituberculates, supporting the assumption that at least in the Jurassic haramiyidans the lower canine was probably lost [20].

### Lower Premolar

The only premolar of *Arboroharamiya* is considered as p4. Its identification as a premolar is based on its morphology, which resembles those in known haramiyidans and some early multituberculates [13], [14], [20]. The lingual side of the left p4 crown is exposed and its tip is broken. The buccal side of the right p4 is exposed but partly broken at its base.

**Figure 3:**
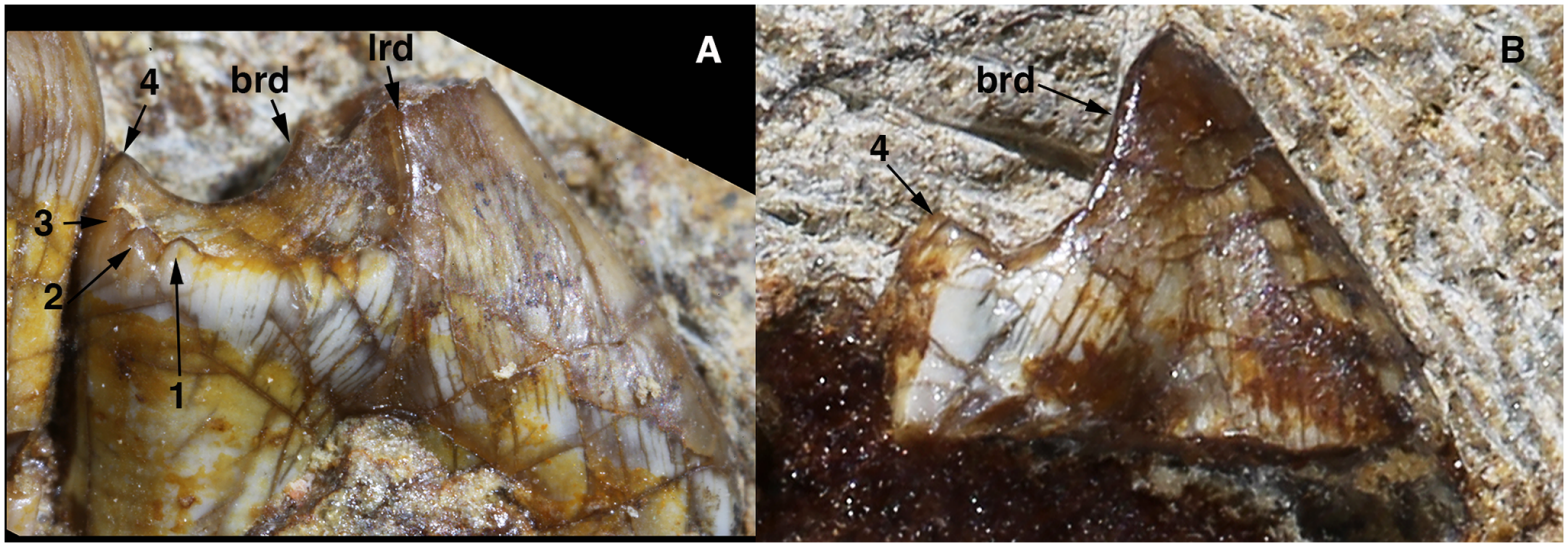
Lower premolar (p4) of *Arboroharamiya jenkinsi.* A, Lingual view of p4. B, Buccal view of p4. Abbreviations: **1–4**, cusps 1–4 on the heel of p4, ranging from the lingual to the buccal side of the tooth; **brd**, buccal ridge on the main cusp of p4; **lrd**, lingual ridge on the main cusp of p4. Note the lack of serrations on both sides of the tooth crown.

It is difficult to assign the p4 cusps using existing terminology because of the variety of the premolar morphologies in haramiyidans. Differing from *Allostaffia* [12], [30], [31] and *Sineleutherus* [14], [15], [32] where two rows of cusps or a series of distinctive cusps are present, the p4 crown of *Arboroharamiya* is predominated by a mesial main cusp and a distal heel bearing several minor cusps. In the description we identify the mesial cusp as the main cusp and refer to the minor cusps with numbers. It is likely that the main cusp is a1. What are numbered as 1–3 are row a cusps, but cusp 4 should belong to row b (Fig. 3).

The p4 has two strong roots, with the anterior one extending anteriorly and the posterior one vertically. It is significantly larger (longer) than m1, with a p4/m1 length ratio of 1.44. The tooth enamel extends to a low position on the anterior surface of the anterior root (Fig. 3). The enamel is thick at the tip and edges of the tooth crown and gradually thins toward the root. Because the two p4s are in the mandibles that are embedded in the slab, the width of the tooth cannot be measured. However, it is clear that the tooth crown is mesiodistally elongate and transversely narrow.

In lateral view, the hypertrophied main cusp has a triangular profile and accounts for more than two-thirds of the p4 length. The apex of the main cusp extends posterodorsally so that its anterior side is much longer than the posterior one. The anterior surface of the main cusp is smoothly convex, bearing no cingulid, cuspules or ridges. This differs from the p4 of Jurassic multituberculates, such as *Kermackodon*, in which the p4 bears at least three serrations [13]. The posterior edges of the main cusp are ridge-like, with the lingual one being more anterior than the buccal one. The posterior surface of the main cusp is flat or slightly concave. The posterobuccal edge of the main cusp continues distally as a crest that defines the buccal border of the heel and terminates posteriorly at cusp 4 (Fig. 3). The posterolingual edge of the main cusp merges with the weak lingual crest of the heel, which is lower than the buccal crest of the heel. The distolingual margin of the heel is decorated with three small cusps, denoted as cusps 1–3 (Fig. 3). These cusps are subequal in size and much smaller than cusp 4. The heel has a shallow concave basin.

Although the p4 of *Arboroharamiya* is mesiodistally elongated, the tooth morphology indicates that the main cusp was used for crushing food, not for cutting or shearing. This matches well with the basin-shaped upper premolars, as will be described below. Among the haramiyidans and early multituberculates for which the lower premolar morphology is known [10], [12], [14], [15], [20], [25], [33], p4 of *Arboroharamiya* is most similar in general shape to that of *Kermackodon* ([13]: Fig. 6B), one of the two earliest known multituberculate genera. However, because the p4 of *Kermackodon* is preserved as an isolated tooth, its orientation is less certain than that of *Arboroharamiya*. Given its current orientation [13], the lingual morphology of p4 of *Arboroharamiya* is more comparable to what has been identified as the buccal side of p4 in *Kermackodon* ([13]: Fig. 6B3). This suggests that the differences in the general tooth plan of the p4 in *Kermackodon* and *Arboroharamiya* are either due to different origins or to the possibility that the orientation of p4 in *Kermackodon* may have been reversed, with the left tooth being identified as the right one.

Among non-multituberculate allotherians, lower premolars preserved in situ were previously known only in *Haramiyavia* [25] and now in *Arboroharamiya* and *Megaconus. Haramiyavia* is morphologically primitive in possessing four premolars, designated as p1-4 [25]; *Megaconus* has two, designated as p1-2 [28]. *Arboroharamiya* is apparently more derived in having only one enlarged premolar, designated as p4. In *Haramiyavia*, the ultimate premolar (p4) is double rooted, larger than the penultimate premolar (p3) and has three tooth cusps that decrease in height distally. A similar morphology is assumed in other haramiyidans [14], [15], [20]. In *Megaconus*, the ultimate premolar (denoted as p2) is single-rooted and much smaller than the double-rooted p1, which is unique among mammaliaforms (see discussion below).

The ultimate premolar is important in the systematics of allotherians. Butler ([19]: 331–332) wrote: “The blade-like (sectorial) p4 is one of the most obvious multituberculate apomorphies, but it may be foreshadowed by *‘Thomasia II*’ and ml of *Haramiyavia,* in which the buccal row is reduced and confined to the distal part of the tooth. If *Thomasia II* is homologous with m1 of *Haramiyavia,* as suggested in this paper, the three molars of that genus could represent p4 to m2 of multituberculates. (It should be noted that the nomenclature of *Haramiyavia* is morphological, without implications of replacement).” In those that have only isolated teeth preserved, identification for some of the premolars remains uncertain. For instance, the *Thomasia II* group teeth were compared with p4 of multituberculates by Sigogneau-Russell [10] and most of them are still considered as the premolars [20]. Butler [19], however, compared *Thomasia* II with ml of *Haramiyavia.* In Eleutherodontidae, to which *Megaconus* is referred to [28], whether those identified as posterior or ultimate premolars (premolariforms) can be compared with the ultimate premolar of *Megaconus* is debatable. In Zhou et al. ([28]: Fig. S5) the premolars of *Sineleutherus* [14], [15] were designated as “left p1”, equivalent to that of *Megaconus.* In our view, however, the tooth listed as “left p1” is most likely the ultimate premolar and is a right tooth.

### Lower First Molar

**Figure 4:**
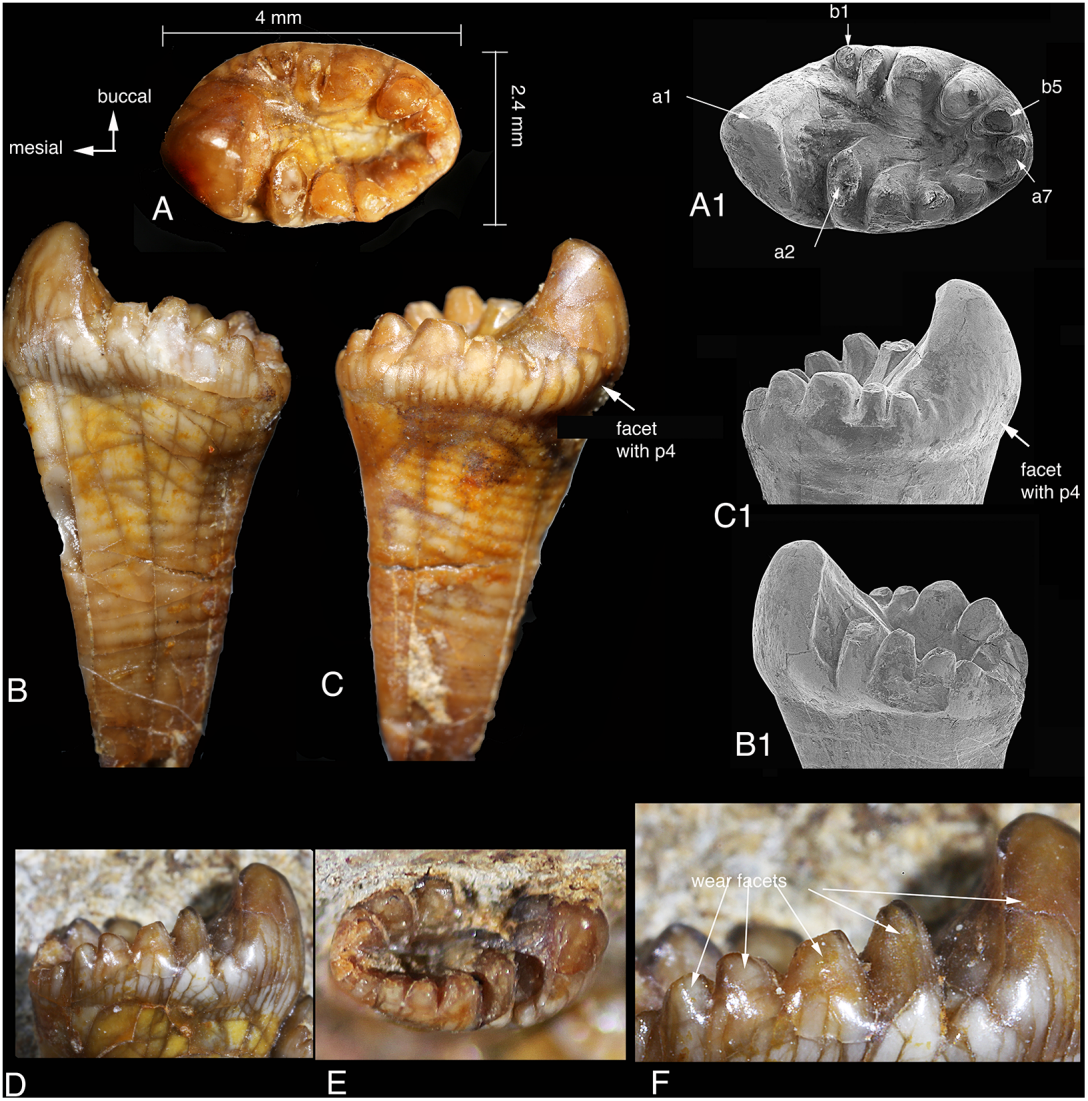
Lower m1s of *Arboroharamiya jenkinsi* (STM33-9). A–A1, Occlusal view of the right m1. B–B1, Lingual view of the right m1. C–C1, Buccal view of the right m1. D, Lingual view of the left m1. E, Occlusal view of the left m1 (slightly tilted). F, Close-up view showing the wear facets on the lingual side of the lingual (row a) cusps. A1–B1 are SEM photographs. All images are on the same scale except for F.

The left m1 is preserved in situ (Fig. 2B); its morphology is identical to that of the right m1, which was separated from the dentary and has been removed from the matrix (Fig. 4). The m1 has a single strong and long root that gradually tapers toward its end. The boundary between the crown and root is discernable but not sharp. There are contour-like lines on the tooth root, perhaps representing growth lines. The lower molar cusps differ from the upper ones in being conical and generally lacking fine enamel ridges (flutings). There is a distinct contact facet on the mesiobuccal side of the right m1 (Fig. 4C–C1), indicating the contact relationship with p4. The position of the facet shows that m1 is not aligned in a straight line with p4, and the relationship of the teeth is illustrated in Zheng et al. ([26]: Fig. 2).

In occlusal view, the outline of m1 is oval or spindle-shaped with blunt ends. As in other allotherians, m1 of *Arboroharamiya* has two rows of cusps. Similar to haramiyidans but differing from multituberculates, m1 cusps are of different heights and sizes and the two cusp rows surround a mesiodistally elongate central basin that is fusiform and deeper distally than mesially, as in *Haramiyavia* [25].

Of the m1 cusps the largest is a1, which is extremely inflated and accounts for about a third of the tooth length, with its apex being situated on the longitudinal axis of the tooth. The anterior surface of a1 is convex and smooth, whereas the posterior surface is largely flat and bears small ridges that extend into the central basin. The lingual and buccal sides of a1 bear wear facets and striations that indicate puncturing and palinal movement of m1.

Row a cusps decease in height and size posteriorly, with cusps a2-7 being significantly smaller than a1. Cusp a2 of the right m1 is broken at its tip, but it is intact on the left m1. Cusp a2 is separated from a1 by a transverse groove. In both m1 s, a2 is unique in being mesiodistally short but transversely wide with its buccal base intruding into the central basin. Cusps a3–a6 are more or less conical. Cusps a6 and a7 at the distal end of row a are significantly smaller than a5 and are confluent due to wear. Their designations are arbitrary, and it is possible that a7 could be counted as b6.

The lingual enamel of the right m1 has been damaged, but the tip of a3 shows wear on its lingual side. The left m1 is intact and shows that there is a distinctive wear facet on the lingual side of each row a cusp (Fig. 4F), which indicates that the lingual row cusps bit into the valley of the upper molar. In addition, the tip of each row a cusp is worn, but the deepest wear is on a6. The a5 and a6 wear facets are confluent and form the floor of the notch at the distal end of the tooth. Moreover, for each of cusps a2–a4 there is a buccal ridge that extends buccodistally; these ridges merge on the floor of the central basin. All of the ridges within the basin have been worn so that they are now flat (Fig. 4A1).

Row b (buccal) has five cusps. Cusp b1 is the smallest and is separated from a1 by a valley that extends mesiobuccally. Cusp b2 is transversely broadened and closely packed with b3, which is the largest row b cusp. The other b row cusps decrease in size and height away from b3 (Fig. 4). Each of the row b cusps bears a wear facet on its tip. In addition, the row b cusps, most distinctively b3 and b4, also have a nearly vertical wear facet on their lingual side toward the basin. No row b cusp bears wear facet on the buccal side.

### Lower Second Molar

**Figure 5:**
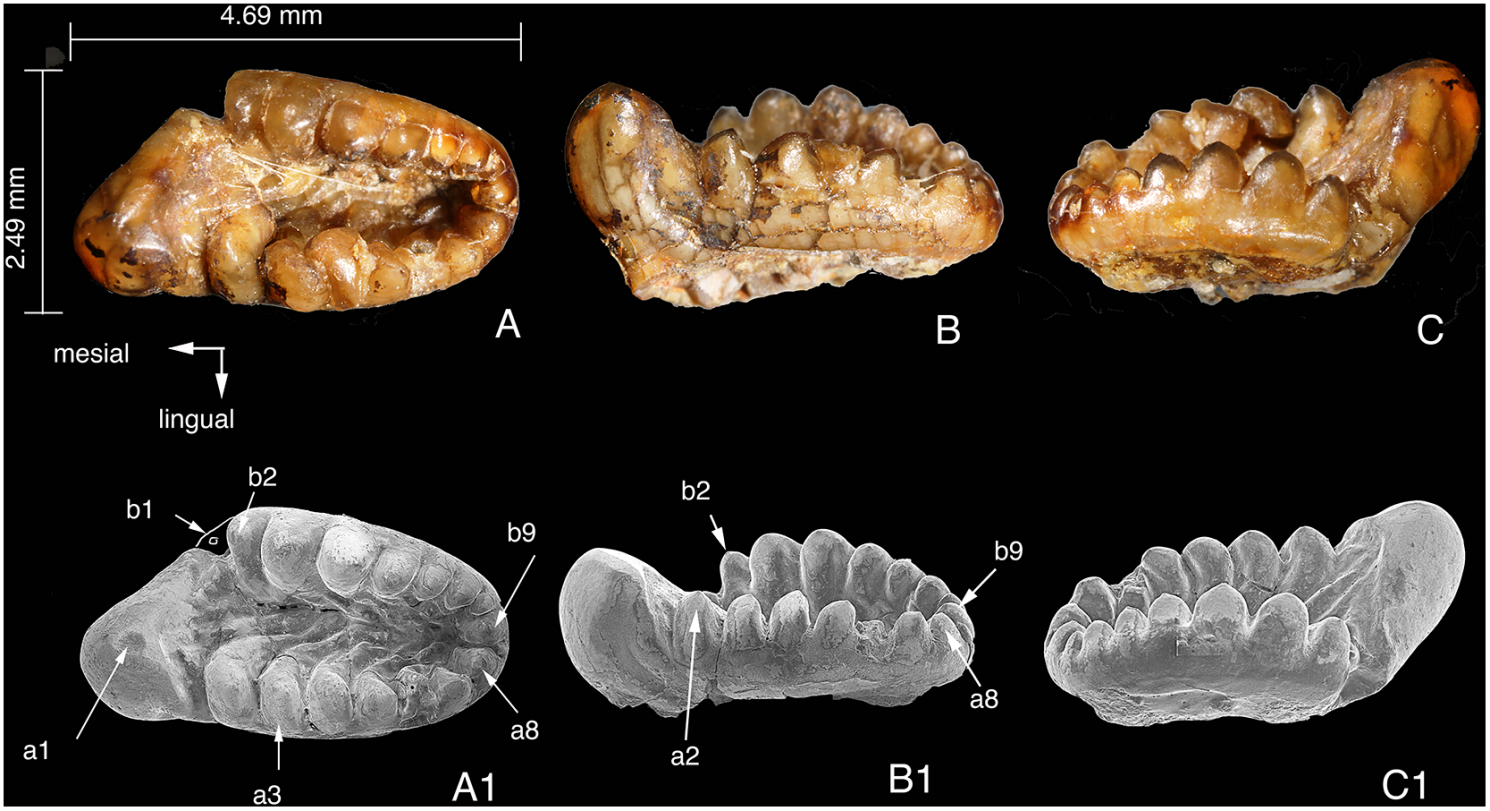
Right m2 of *Arboroharamiya jenkinsi* (STM33-9). A–A1, Occlusal view; B–B1, Lingual view. C–C1, Buccal view. Note the breakage at b1. Note cusp a3 and a4 are slightly displaced in A–C, but restored to their anatomical positions in A1–C1. A1–C1 are SEM photographs. All images are on the same scale.

Immediately posterior to the left m1 is the alveolus for m2, as preserved in the left dentary (Fig. 2B). By its position, m2 is lingual to the coronoid process so that m2 is not visible in buccal view of the mandible, a feature similar to multituberculates. The left m2 is still embedded in the slab and is partially broken. The right m2 crown is preserved in good condition and was removed from the slab during preparation (Fig. 5). The root is not preserved in either m2. The m2 is larger than m1 and has eight row a and nine row b cusps, more than those of m1.

Similar to m1, a1 is greatly inflated, but it is more lingually situated in line with other row a cusps so that the mesial end of the tooth protrudes mesiolingually and that the mesiobuccal side of cusp a1 is longer than that of m1. The a1 is more blunt, more procumbent and shorter, than that of m1. There are wear facets and striations on the lingual and buccal sides of a1 and on the low ridges on the posterior surface of a1.

Similar to that of m1, the a2 cusp of m2 is transversely broadened, with its buccal side extending into the central basin. Row a cusps decrease in size distally. Cusps a3-4 were fractured and slightly displaced (Fig. 5A–C) but they have been restored back to their original positions (Fig. 5A1–C1). Cusp a6 is damaged. Compared to row b cusps, row a cusps have a tendency to be more transverse and to have cusp tips curving distally. On the buccal side of a2–a6, each cusp extends distobuccally to the central basin as a distinct ridge.

In row b of m2, the area bearing b1 is broken, creating a notch mesial to b2. Cusp b1, if preserved, must be small. Similar to m1, b3 is the largest row b cusp in occlusal view, but b4 is only slightly thinner but is taller than b3. Cusp b8 is the smallest on row b, smaller than b9. Compared to the buccal sides of row a cusps, the lingual sides of row b cusps are steeper. Similar to row a cusps but in a distolingual direction, the lingual side of each row b cusp also continues into the floor of the central basin as a ridge. These ridges are steeper and shorter than those of row a cusps. The basin floor is thus full of enamel ridges that are sharp and must have functioned as cutting edges analogous to those of a rasp. Apparently these ridges form a mechanism for grinding food particles when the lower molars move palinally with the upper tooth cusps biting in the basin.

The central basin is significantly longer and deeper than that of m1 and is closed distally by cusps instead of a ‘U-ridge’ that is present in other haramiyidans [13], [19], [20]. The posterior end of the basin is so deep that if any upper tooth cusp is in the basin it is absolutely impossible for the lower jaw to move mesially. This basin morphology and the wear facets on cusps of lower molars demonstrate that the lower jaw of *Arboroharamiya* is capable of orthal and palinal movements, but certainly not for proal and transverse chewing motion.

It is also clear that, unlike m1 cusps, there is no wear on m2 cusps except for a1. The ridges on the basin floor are still sharp, similar to the unworn cusps of M2 (see below). These suggest that M2/m2 erupted at a later stage of ontogeny.

### Upper Incisor

**Figure 6:**
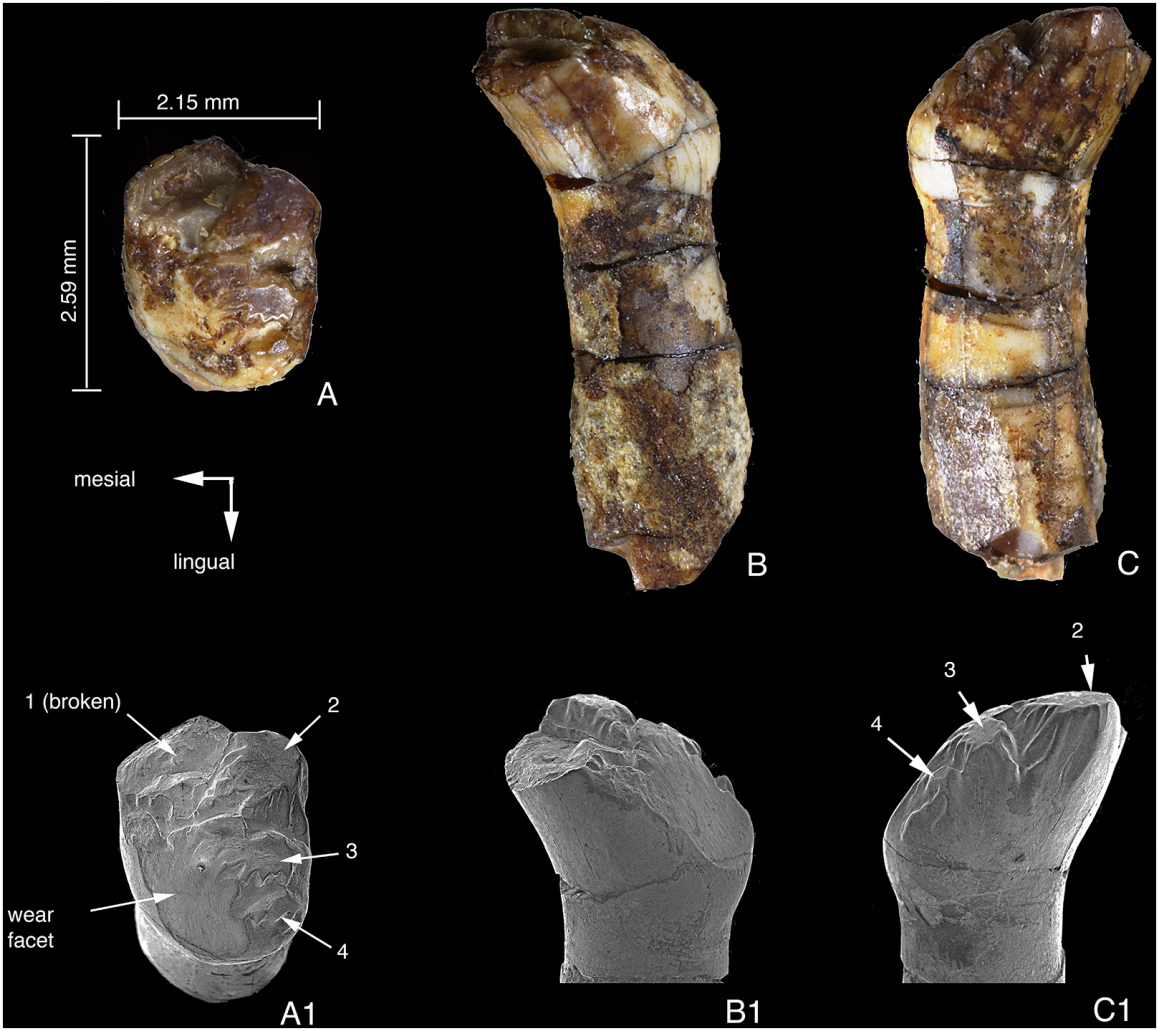
Left upper incisor of *Arboroharamiya jenkinsi* (STM33-9). A–A1, Occlusal view; B–B1, Mesial view. C–C1, Distal view. A1–C1 are SEM photographs. All images are on the same scale.

The upper incisor is considered as I1 [26], although it may well be I2 compared to the enlarged I2 in multituberculates. This may be verified when new specimens with better preservation become available. The upper incisor is single-rooted, and the root is long and strong. Unlike the lower incisor, I1 crown is not elongated but is multi-cuspate, as in some multituberculates. Based on its morphology and wear facets we identify it as the left incisor. The tooth crown consists of four cusps that are decorated with small ridges, mostly on their distal sides. Because of these ridges, the occlusal outline of each cusp becomes complex after wear. The largest cusp is the mesiolingual one (cusp 1 in Fig. 6); it was broken, but its posterior base and the buccal border are clear enough to infer its size. From the breakage it can be determined that the incisor enamel is thin. Cusp 2 is buccal to cusp 1 and slightly smaller than the latter. The cusp bears a flat wear facet at its tip. The preserved parts of cusps 1 and 2 nearly account for the anterior half of the crown length.

Cusp 3 is situated on the distal side of cusp 2 and is also heavily worn. This cusp is transversely extended. The distal cusp (cusp 4) is tiny but still bears a wear facet on its tip. In addition to the wear on the cusp tips, there is a large wear facet on the distolingual side of the tooth, confluent with the lingual side of cusp 3. This wear facet has a high angle. Judging from the two kinds of extensive wear facets this tooth must be the functional upper incisor. Whether there are additional incisor(s) between this tooth and the premolar is unknown, but it is probable that there is only one upper incisor.

### Upper Third Premolar

**Figure 7:**
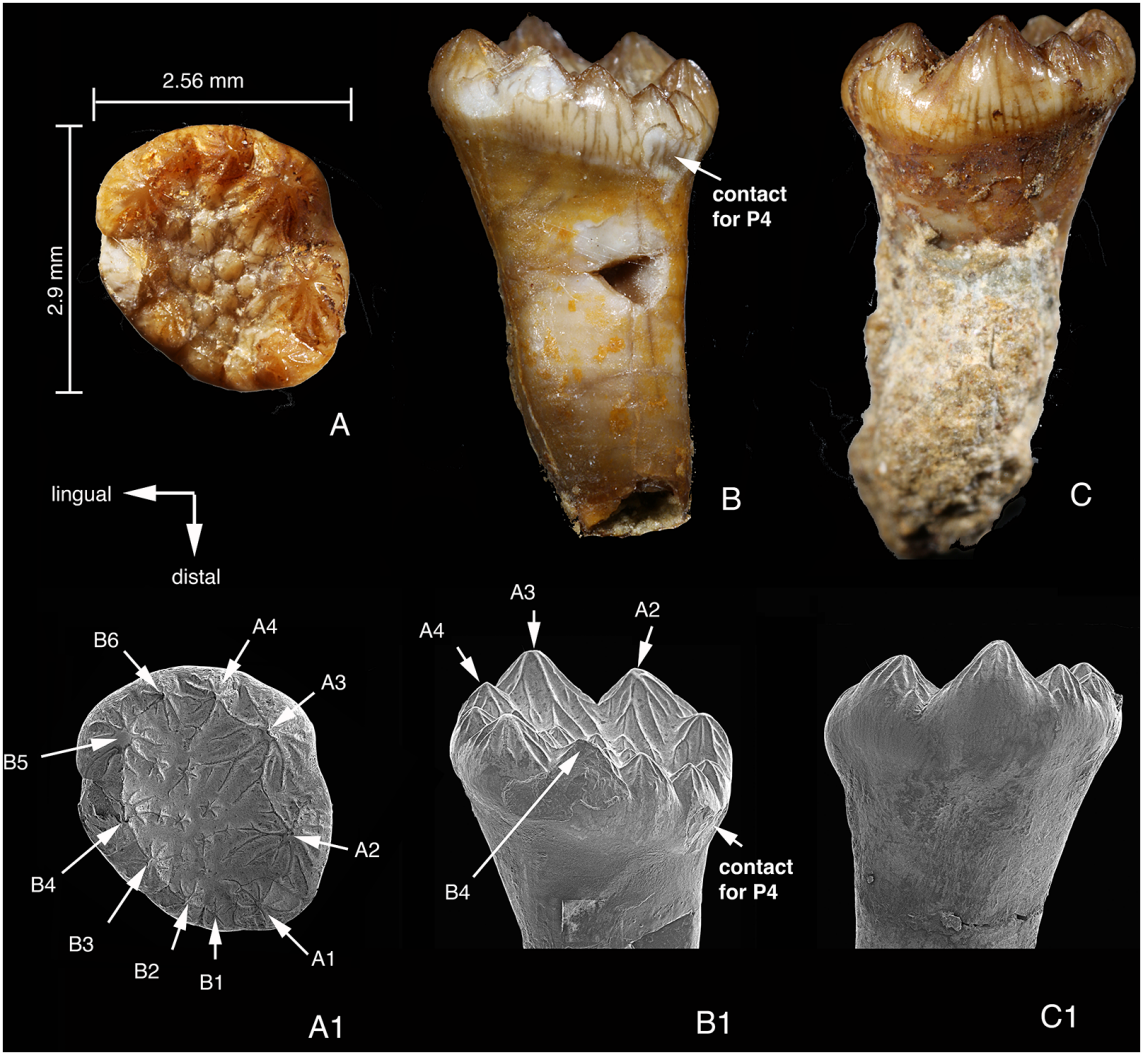
Left P3 of *Arboroharamiya jenkinsi* (STM33-9). A–A1, Occlusal view; B–B1, Lingual view. C–C1, Buccal view. A1–C1 are SEM photographs. All images are on the same scale.

Among the preserved upper cheek teeth, the tooth interpreted as the left P3 is the smallest (Fig. 7). The tooth has a long, strong root. The anatomical orientation of the tooth is interpreted such that the crown is more mesial than the end of the root. In addition, the contact facet for P4 at the distal end of the crown is also distinctive. In such an orientation the root is transversely narrow and would extend dorsodistally in the maxilla. Given that the largest cusp is distobuccal in P4 and M1, row A and row B cusps of P3 can be determined by their sizes. This orientation also makes sense in that small B1-2 form a low distal border for the P3 basin that allows the palinal motion of p4 during chewing.

P3 is much smaller than P4, but they share similar morphologies in two aspects. First, the occlusal outline of the tooth is oval and the main cusps are located peripherally, surrounding a shallow broad central basin. Second, all cusps, large or small, are conical and ornamented with minor ridges or flutings [19], [34] that radiate from the tip of the cusp. These features distinguish the upper premolars from the upper molars and lower check teeth. In contrast the upper molar is mesiodistally elongate and roughly rectangular. The morphology of P3 suggests a function for crushing food.

Given the tooth orientation, row A consists of four cusps with A3 being the largest and row B has six cusps with B5 being the largest. In the central basin there are several small cuspules. Unlike P4, the cusps in the basin do not bear any wear. This implies that P3 was not yet used and that it erupted later than P4, perhaps at the same time when m2 erupted. If this is the case, then P3 in this individual is probably a successive tooth.

### Upper Fourth Premolar

**Figure 8:**
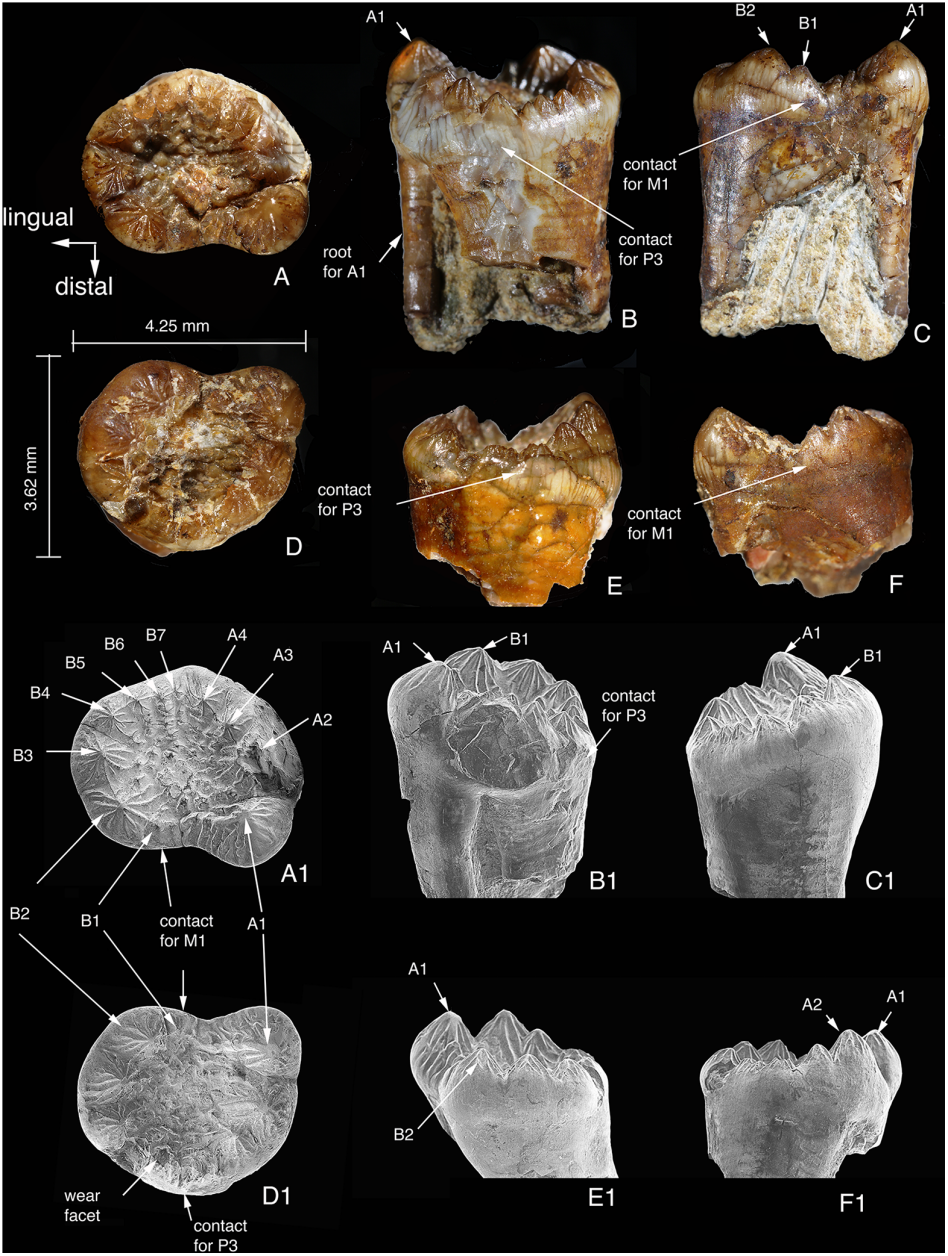
P4s of *Arboroharamiya jenkinsi* (STM33-9). A–A1, Occlusal view of the left P4; B, Mesial view of the left P4; B1, Mesiobuccal view of the left P4; C, Distal view of the left P4; C1, Lingual view of the left P4. D–D1, Occlusal view of the right P4; E, Mesial view of the right P4. E1, Lingual view of the right P4. F, Distal view of the right P4. F1. Buccal view of the right P4. A1–F1 are SEM photographs. All images are on the same scale.

The right and left P4s are preserved and were removed from the slab during preparation (Fig. 8). Because the lower dentition has only three cheek teeth, we assume that the upper dentition has only four cheek teeth in *Arboroharamiya*. The two P4s mirror each other in morphology. The left P4 crown is preserved in a better condition. Similar to P3, all cusps are conical and ornamented with flutings except for those that were deeply worn.

It has been known that the distal cusps are the largest in the upper cheek teeth of haramiyidans [19], and this is true in *Arboroharamiya.* In addition, the contact facets on P4 help to determine the mesial and distal ends of the tooth (Fig. 8). The distal margin of the tooth is broadly concave, which receives the convex mesial end of M1. The left and right sides of P4 can be determined by the root condition. The right P4 has a strong root that is fused proximally by three smaller roots: a major lingual root and two minor labial ones. The distal tips of these roots are separated. The largest cusp is the distobuccal A1, which is supported by a small distobuccal root. Cusp row B (lingual) is supported by a robust lingual root that is fused with the mesiobuccal root. The root condition is similar to the common condition of other mammals in which the lingual crown is usually supported by a main root and the buccal side by two separate minor roots. Considering the orientation of the tooth, it is clear that the largest cusp is the distobuccal one (A1). The concave distal edge of P4 fits well with the convex mesial end of M1 (see below).

The width of P4 is greater than its mesiodistal length. Row A has four cusps, with A1 being the largest, A4 the smallest and A2 and A3 being subequal. There are seven cusps on row B. Cusp B1 is small and B2 is the largest; other cusps decrease in size mesially from B2. The mesial side of the central basin is closed by cusps, which contrasts with the open distal end between A1 and B1. Again, this morphology allows palinal movement of the lower jaw. The central basin is broad and has numerous small cusps, some of which in the central area bear wear on their tips (Figs. 8, 12D, J), created by contact with the lower premolar in orthal chewing. The only wear that is not in the central basin is near the mesial margin of the tooth (Figs. 8D1, 12J).

### Upper First Molar

**Figure 9:**
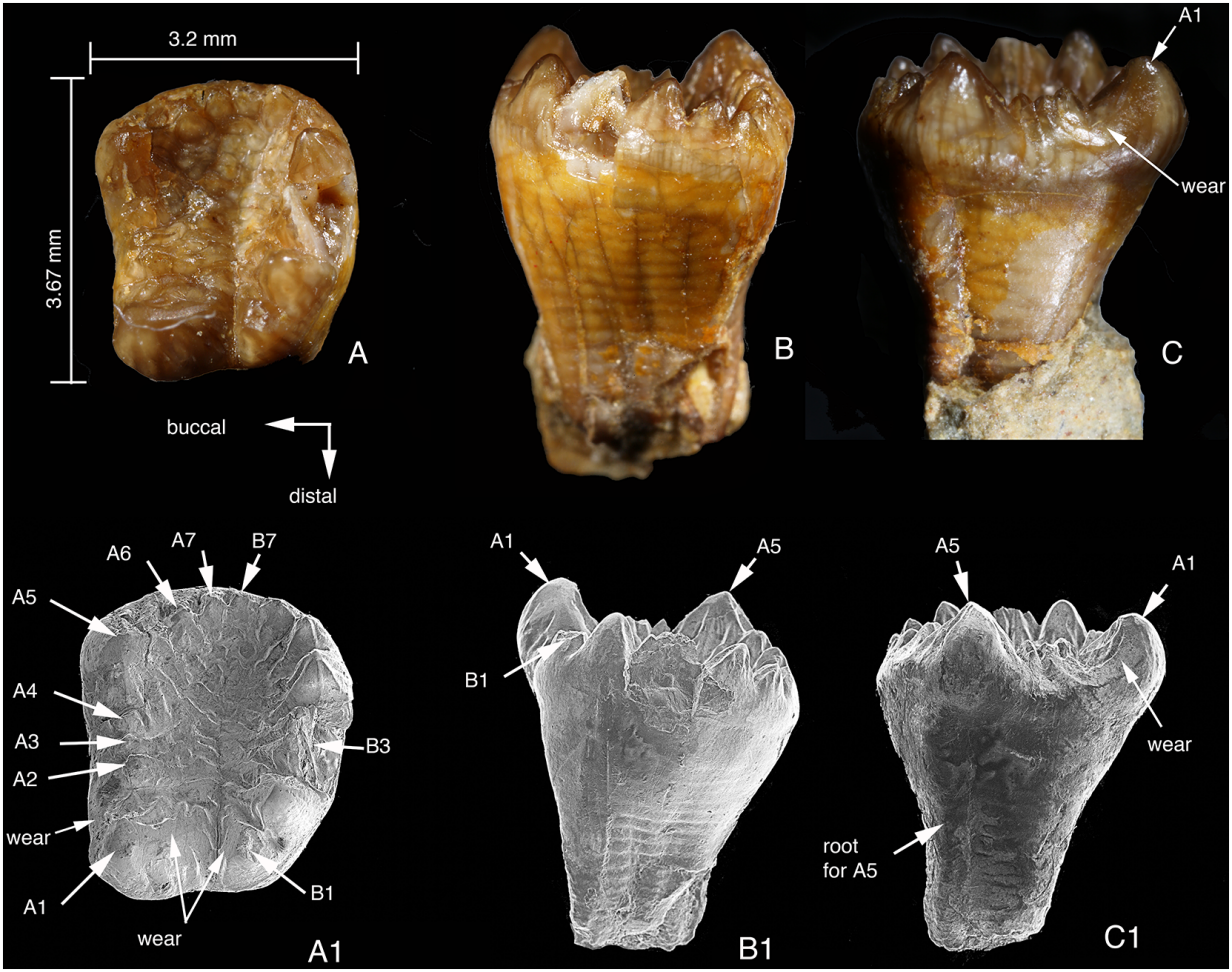
Right M1 of *Arboroharamiya jenkinsi* (STM33-9). A–A1, Occlusal view. B–B1, Lingual view. C–C1, Buccal view. A1–C1 are SEM photographs. All images are on the same scale.

The occlusal outline of M1 is roughly rectangular (Fig. 9) with its length being consistent with that of m1. The anterior half of the tooth is slightly wider than the posterior, and the mesial edge of the tooth is convex. On the anterior surface of the tooth, along A6-7 and B6-7, there is a continuous contact facet for P4. This contact area fits into the broad bay at the distal end of P4, which probably helped to stabilize the teeth but not identically to the interlocking mechanism seen in some other mammals [28].

The tooth crown is supported by a strong root that becomes thinner distally. The mesiobuccal cusp (A5) is supported by a small root, which is fused proximally to the rest of the root. The distal tips of the roots are still separate. A vertical groove along the root delimits the two parts (Fig. 9C–C1). This condition is similar to what was termed as “the hypsodont and proximally fused multiple roots” [28]. On the mesial surface of the crown exists the contact facet for P4. Its perfect match in occlusion with the right m1 and see later discussion) can also be used to support the orientation of M1.

It was noted that in other haramiyidans [19], [20], the central basin of the lower molar is closed distally by a “U-ridge” but open mesially, whereas the basin condition of the upper molar is reversed, with the mesial end being closed and the distal end open. The same feature exists in the molars of *Arboroharamiya.* The distal opening of the M1 central basin is a V-shaped notch in distal view and the mesial edge of the tooth is closed by a series of small cusps. The cusp arrangement is not distinctively in two rows, rendering Butler’s [19] cusp terminology arbitrary (Fig. 9A1). Nonetheless, the cusps are divided into A and B rows, and there is no extra row C buccal to row B, a condition present in other haramiyidans such as *Haramiyavia* [25].

A1 and A5 are subequal in size and are the main cusps in row A. Between A1 and A5 there are three small cusps denoted as A2-4. The mesial side of A1 has three enamel ridges that extend to the basin floor and the lingual ridge bears wear facets (Fig. 9A1). The buccal side of A1 also bears an extensive wear facet, which extends to the buccal sides of A2-3 (Fig. 9C1). This wear pattern indicates that cusp A1 must have bitten into the central basin of the lower molar so that its lingual and buccal sides were in contact with cusps of the lower molar.

The three small cusps (A2-4) are closely packed and transversely wide; each of them continues mesiolingually as a ridge extending to the basin floor. A5 has a convex mesiobuccal surface and has wear on its ridges that extend distolingually to the basin. There is no wear on the buccal side of A5, a condition similar to that of *Eleutherodon oxfordensi* [19], [34], except that the tooth orientation is reversed (see below). Two small cusps on the mesial edge of the tooth crown are counted as A-row cusps and denoted as A6 and A7.

Row B consists of seven cusps, of which B3 (damaged) is the largest one. B-cusps decrease in size both mesially and distally away from B3. B4 and B5 bear flutings. The mesial end of the central basin is closed by A6-7 and B6-7. The distal sides of these four small cusps bear wear. The wear facets on the buccal sides of B1 and B2 (Fig. 9A1) are more extensive and striations on these wear facets show mesiodistal movement of the lower teeth. There is no wear on the lingual side of the B-cusps.

The central basin is broad and full of enamel ridges (flutings) that are extended from the cusps. These ridges have different thickness and, after wear, display a complex pattern. The degree of wear in M1 matches that of m1. There is a narrow groove that runs longitudinally, separating the posterior half of the tooth basin into two parts and terminating distally in the V-shaped notch between cusps A1 and B1.

### Upper Second Molar

**Figure 10:**
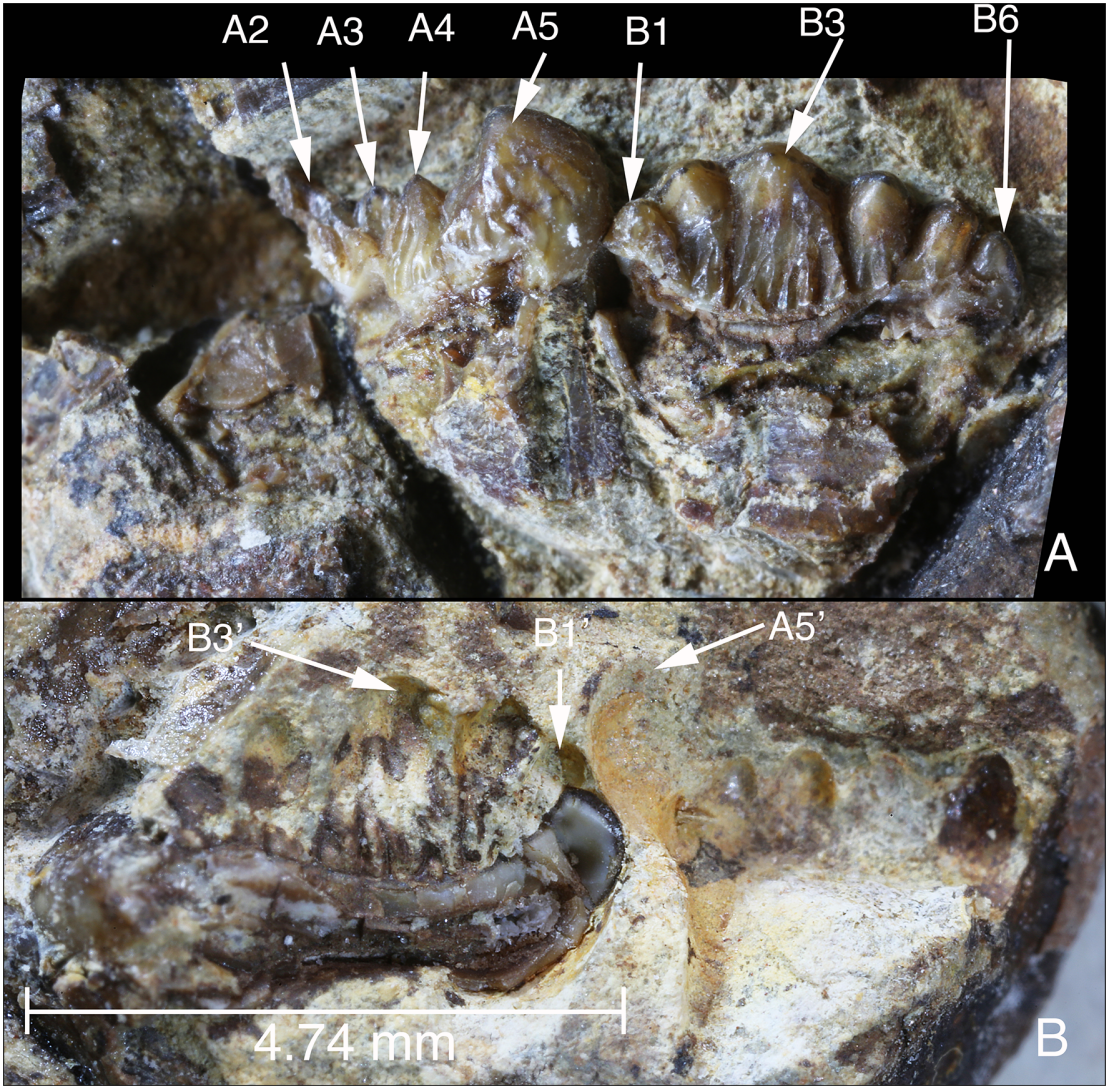
Partial M2s of *Arboroharamiya jenkinsi* (STM33-9). A, Occlusal (tilted) views of the buccal part of left M2 with cusps A2–A5 and lingual side of right M2 with cusps B1–B6 preserved in slab a. B, Impressions of cusps in A preserved in the counterpart of the slab. The buccal half of the right M2 is still in the matrix of the counterpart. A5’, B1’ and B3’ are impressions of the corresponding cusps in A. Images are on the same scale.

The buccal part of the left M2 with cusps A2–A5 and lingual part of the right M2 with cusps B1–B6 are preserved in slab a (Fig. 10A). The impressions of these cusps and the rest of the buccal half of the right M2 are preserved in the counterpart slab (Fig. 10B).

The buccal part of the left M2 shows a morphology similar to that of the right M1, such as the conical mesiobuccal cusp (A5) and the three small cusps (A2-4) distal to A5. Similarly, B3 is the largest cusp among row B (lingual) cusps of the right M2.

M2 differs from M1 in its larger size. The outline of the longitudinal cross section of the left M2 in slab A is transected by a crack in the matrix, but the cross section of the tooth is clear and unaltered in slab B, allowing an accurate measurement of the length. The M2 length is 4.75 mm, consistent with that of m2 (4.7 mm) but significantly longer than M1 (3.67 mm). In addition, M2 also differs from M1 in some aspects of its detailed morphology. The three small cusps (A2-4) are proportionally larger than those in M1. M2 cusps, particularly B3, bear fine and distinct ridges (flutings) that show little wear, matching the wear state of m2.

### Occlusal Relationships and Wear Patterns

**Figure 11:**
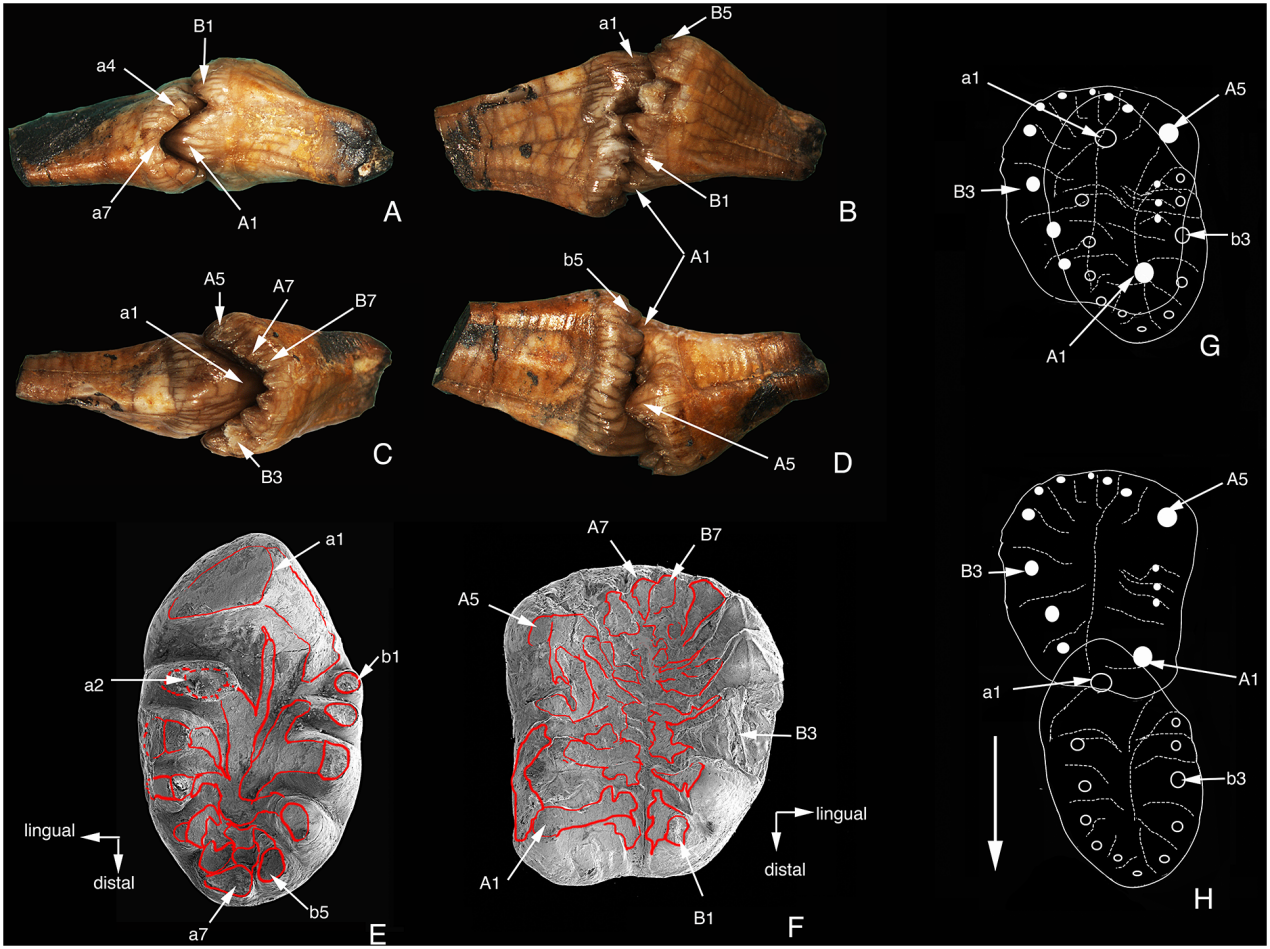
Occlusal and wear pattern of *Arboroharamiya jenkinsi* (STM33-9). A–D, Distal, lingual, mesial and buccal views of the right M1 and m1 in occlusion. E, Occlusal view (SEM photograph) of the right m1 with wear facets outlined with red lines. F, Occlusal view (SEM photograph) of the right M1 with the wear facets outlined with red lines. G, Diagram showing the tooth cusp relationships of M1 and m1 in occlusion. H, Diagram showing the tooth cusp relationship of M1 and m1 when m1 moves distally at the end the palinal cycle of the chewing motion. A–D and G–H are modified from Zheng et al. [26]. A new arboreal haramiyid shows the diversity of crown mammals in the Jurassic period. Nature 500: 199–202 (DOI: 10.1038/nature12353). Reproduced by permission of Nature Publishing Group. Images are not on the same scale.

The right m1 and M1 from the same specimen of *Arboroharamiya* are preserved in good condition and are large and strong enough to allow manipulation to illustrate cusp relationships in occlusion (Fig. 11). When fitting the two teeth together, there is only one matching occlusal position, as shown in Figure 11A–D. This position most probably represents the beginning stage of the palinal cycle during chewing. At this stage, the hypertrophied a1 of m1 occluded with the mesial part of the M1 central basin (Fig. 11C), whereas A1 of M1 was positioned in the distal end of the central basin of m1 (Fig. 11A). This occlusal relationship was termed “double engaged” [26]. The relationship is also illustrated in Figure 11G. In addition, the distal view of row a cusp of m1 is seen at the notch between A1 and B1 of M1 (Fig. 11A). Row b of m1 is located buccal to A2-4, and A5 of M1 is mesial to the gap between a1 and b1 of m1. When the lower and upper molars occlude in such a relationship, it is impossible for the lower jaw to move either proally or transversely. From this position the lower jaw can only move palinally.

Figs. 11A–D and G represent only one position of the chewing motion. It can be inferred from the wear markings that before reaching the illustrated position, a1 must have been in contact mesially with the distal sides of A7 and B7 to create wear on those cusps. Similarly, A1 must have been distally in contact with a6-7 to create wear on those cusps. In such an occlusal relationship as illustrated in figures 11A–D and G, the orthal and palinal motions of the lower molar will create wear facets at certain areas of both upper and lower teeth. For m1, wear facets are present on the lingual and buccal sides of a1, on tips of all cusps, on the lingual side of row a (lingual) cusps, and on the ridges in the central basin. The buccal sides of the row b cusps are not worn. For M1, wear facets are present on the basin sides of all cusps as well as on the buccal sides of A1-4. There is no wear on the lingual side of any row B cusps. The wear facets illustrated here and in Figure 4F indicate that it is the lingual cusps, most importantly a1, that occluded with the basin of the upper molar and then moved palinally during mastication. The lingual side of row a cusps must have been in contact with the buccal side of row B cusps of the upper molar during chewing to create the wear facets on the lingual side of row a cusps. The tooth morphology and wear patterns also show that it is impossible for the buccal row (row b) of m1 to have occluded with the valley between A and B rows of M1 and for the lingual row of M1 to have occluded with the valley of m1, which is a common view held for occlusion of haramiyidans [11], [13], [17]–[20].

**Figure 12:**
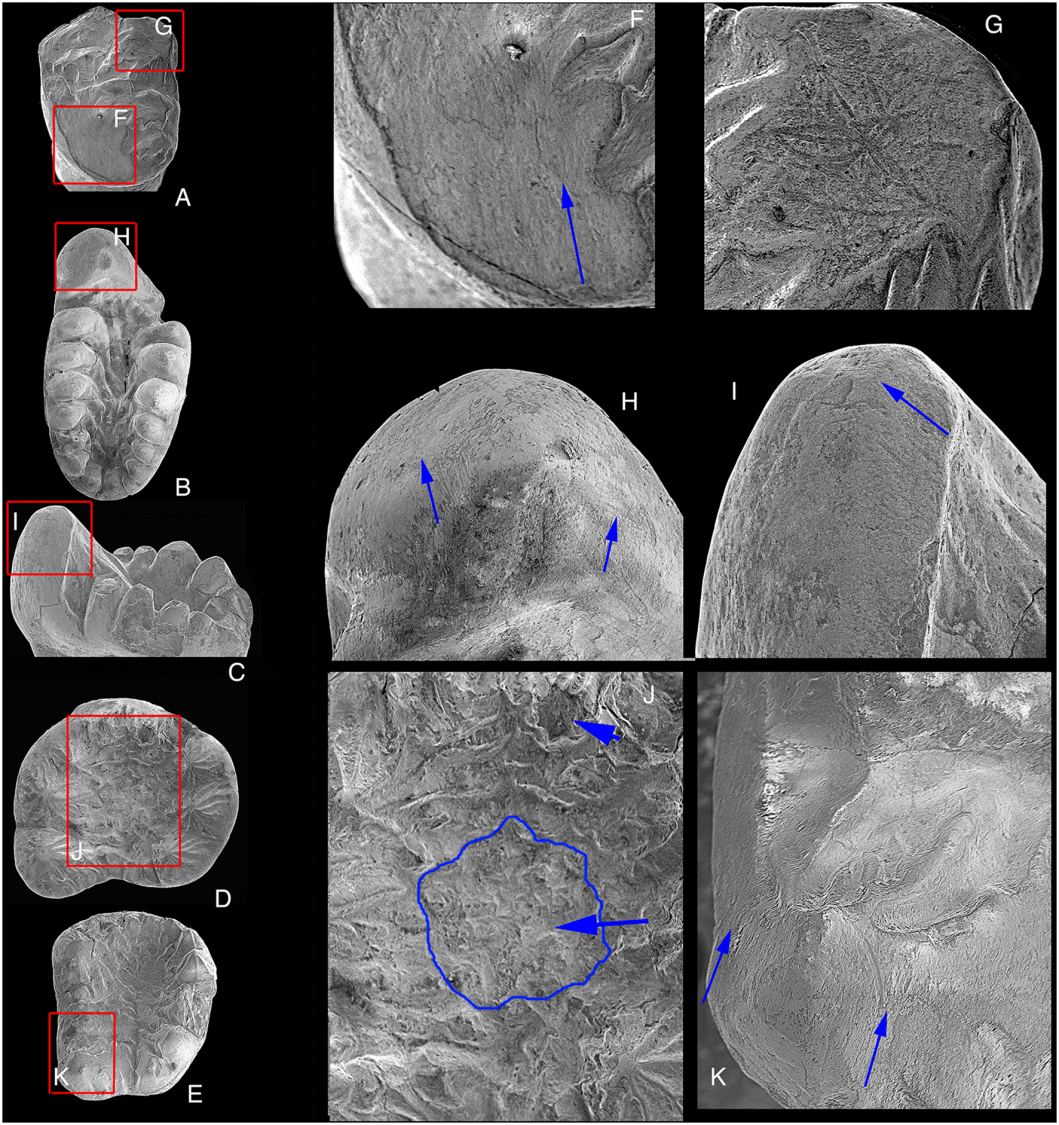
SEM photographs showing tooth microwear of *Arboroharamiya jenkinsi* (STM33-9). A, Crown view of the upper left incisor. The bed boxes G and F correspond to the close-up images in G and F. B, Crown view of the right m2, with the red box H corresponding to the close-up image H. C, Lingual view of the right m1 with the red box I corresponding to the close-up view in I. D, Crown view of the right P4 with the red box J corresponding to the close-up view J. E, Crown view of the right M1 with the red box K corresponding to the close-up view in K. Blue arrows in J indicate worn cusps in the tooth basin of P4. Blue arrows in F-I and K indicate the directions of striations on the wear facets. Images are not on the same scale.

The flat wear facet on the tip of cusp 2 (on cusps 3 and 4 as well) of the upper incisor does not show any directional striations, suggesting that the tooth has been used to pick up food in life (Fig. 12). The striations on the wear facet at the distolingual side of the tooth are nearly parallel to the mesiodistal axis of the tooth crown, which indicate shearing motion of the incisor. The directions of the striations on the lingual and buccal sides of a1 of m1-2 (Fig. 12H, I) indicate that a1 occluded with the valley of the upper molar and moved palinally during chewing. The wear on the small cusps in the basin of P4 (Fig. 12J) must have been created by contact with the main cusp of p4 during the puncturing or crushing cycle of the mastication. Moreover, the direction of the striations on the lingual and buccal sides of A1 of M1 (Fig. 12E, K) indicates that A1 occluded with the valley of the lower molar and the latter moved palinally during chewing.

The occlusal relationship and tooth wear pattern of *Arboroharamiya* are unique among mammals, differing from that of multituberculates and from what has been interpreted for haramiyidans [11], [13], [19], [20]. However, in multituberculates, even in the early forms, such as the Paulchoffatiinae [35], [36], the occlusal relationship of M2/m2 are similar to that of *Arboroharamiya* in that the lingual cusps of m2 occluded with the central valley of M2 and the buccal cusps of M2 occluded with the valley of m2.

## Comparison

Butler [19] considered that the order Haramiyida contains two suborders and four families: Theroteinidae in Theroteinida [2] and Haramiyidae, Haramiyaviidae and Eleutherodontidae in Haramiyoidea [7]. This classification was followed by Kielan-Jaworowska et al. [4]. We will give a brief comparison of *Arboroharamiya* with the four families and then in more detail with *Megaconus* whose placement in Eleutherodontidae [28] we here question.

### Theroteinidae

Theroteinidae is the only family in Theroteinida [9] and was diagnosed “with fully orthal occlusion, in which upper and lower molars alternate, so that each lower molar bites against two upper molars. The highest cusps are more centrally placed on the teeth than in Haramiyoidea, and the longitudinal valleys are short.” In addition, the family is further diagnosed in having short and wide upper molars, with an additional lingual row of cusps, and cusps being low and obtuse [19].

None of the features are applicable to *Arboroharamiya*. Apparently, *Arboroharamiya* is morphologically more derived in being larger and having two rows of cusps in upper and lower molars with hypertrophied a1 and A1. The central basin in the upper and lower molar is mesiodistally long and deep, and the chewing motion consists of a significant palinal component.

### Haramiyaviidae

Haramiyaviidae, Haramiyidae and Eleutherodontidae were placed in the suborder Haramiyoidea [7]. They shared the following features ([19]: 334): “The lower molars are nearly opposite the upper molars, so that there is only transient contact with the more anterior upper molar. The median valley is longer than in Theroteinida, occupying most of the length of the tooth, except on anterior lower molariforms (unknown in Eleutherodontidae), where it is confined to the distal part of the tooth. Palinal occlusal movement developed to various extents; it is incipient in Haramiyaviidae and most extensive in Eleutherodontidae”.

The family Haramiyaviidae [19] is based exclusively on *Haramiyavia* [25]. The diagnostic features, modified from Jenkins et al. [25] and Butler [19], include upper molars wide (subcircular) with additional cusps on the buccal side. On lower molars the highest cusp is a1 and the second highest is b2. The longitudinal valley of m1 is confined to its distal part. Palinal occlusal movement was probably short. Lower premolars have a single row of cusps. Three upper incisors are unspecialized and equal in size. The lower dentition has four lower incisors, a single-rooted canine and four premolars. The dentary possesses a trough for postdentary bones (see discussion below) and the masseteric fossa does not extend forward below the molars.

There are many features that differentiate *Arboroharamiya* from Haramiyaviidae or *Haramiyavia.* Most distinctively, *Arboroharamiya* has a reduced dentition with only one lower incisor, no canine, one premolar and two molars in the lower dentition; the upper dentition probably has one incisor, no canine, two premolars and two molars. The molars are mesiodistally long and surrounded by many more cusps. The premolars have distinct flutings and the tooth basin is broad. The mandible is robust and deep, with the masseteric fossa extending anteriorly below p4. However, *Arboroharamiya* is similar to *Haramiyavia* in having a1 as the largest lower cusp and the lower molar basin deeper distally than mesially.

### Haramiyidae

Haramiyidae [37] differs from Haramiyaviidae in having upper molars longer than wide, with two rows of cusps, the supplementary buccal cusps absent or represented by a cingulum. The basin is closed distally by a ridge (‘saddle’) between the buccal and lingual cusps. On lower molars the first buccal cusp is rudimentary or absent. Except on the anterior molariform, the highest (second) buccal cusp is directly opposite the first lingual cusp and joined to it by a saddle that closes the basin mesially. Palinal chewing, in which cusps moved longitudinally in the basin of the opposing tooth, was well developed. Anterior upper molar more narrowed mesially, with lingual cusps confined to the distal part of the tooth. Referred upper incisors differentiated: I2 enlarged, with distal basal heel [19].

The main member of Haramiyidae is *Thomasia* Poche, 1908 (including *Haramiya* Simpson, 1947). *Arboroharamiya* differs from *Thomasia* in having the central basin of upper molar open distally but closed mesially by several small cusps. M1 is wider mesially than distally. The buccal row cusps A1 and A5 are distinctively larger than those between them. On the lower molars there are more cusps surrounding the elongate central basin. The first buccal cusp is small and b3 is the largest b-row cusp. The highest cusp is a1, which is hypertrophied and extends mesially. Both orthal and palinal chewing motions were developed.

### Eleutherodontidae

Eleutherodontidae [34] was diagnosed by Butler ([19]: 335) as: “Haramiyoidea with upper molars wide, rhomboidal in outline and possessing three rows of cusps. The additional row is lingual and occludes lingually to the lower molar (a character shared with Theroteinidae, probably by convergence). Lower molars oval, with two rows of cusps that are continuous round the distal end. The largest cusps are at the distal end of the middle row on upper molars, and at the mesial end of the buccal row on lower molars; also the mesial upper lingual cusp is enlarged. Minor cusps are numerous and variable. The longitudinal groove of upper molars, between the buccal and middle cusp rows, extends the whole length of the tooth; it is not interrupted by a saddle as in Haramiyidae. Palinal occlusion was extensive, but retained an orthal component as in Haramiyidae. Upper molar has a shorter and shallower groove, between the middle and lingual cusp rows, for occlusion with lower lingual cusps. The sides of the occlusal grooves of upper and lower teeth are covered with numerous minor transverse ridges (‘fluting’). Eleutherodontidae differ from all other Haramiyida in: the more numerous cusps (e.g. there are up to ten upper buccal cusps); the anterior position of the large lower buccal cusp, which projects mesially beyond the lingual row; and the fluting”.

Here we cite Butler’s [19] diagnosis for Eleutherodontidae for two reasons: First, as discussed below in “Tooth orientation and occlusion of haramiyidans”, we demonstrate that the tooth identification in previously known eleutherodontids has been reversed, that is, the left tooth was identified as the right one. Second, the diagnosis for Eleutherodontida has been amended by Zhou et al. [28] because of the inclusion of *Megaconus*. However, we question the taxonomic placement of *Megaconus*, as we will detail below.

**Figure 13:**
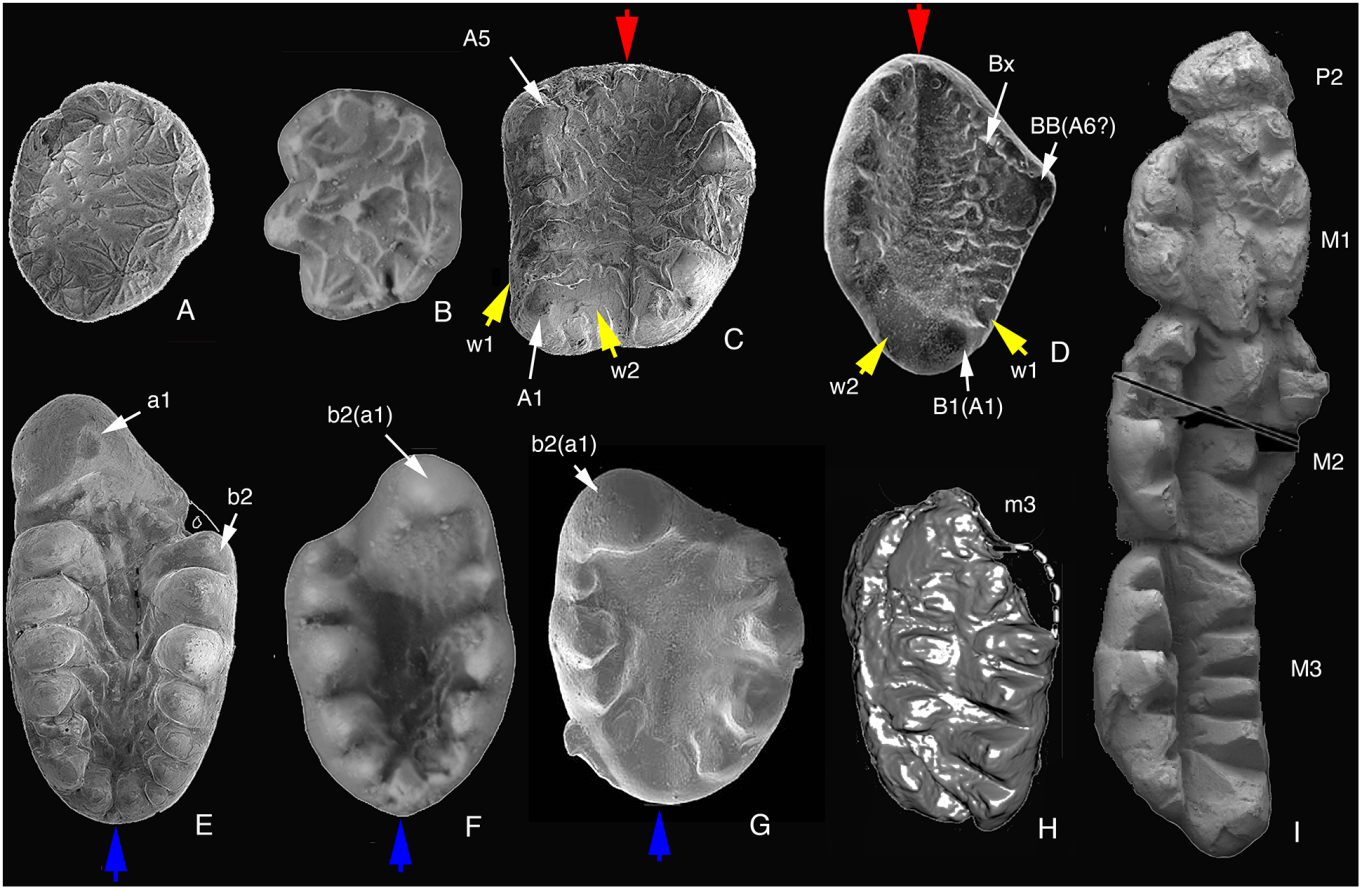
Tooth comparison between *Arboroharamiya jenkinsi* and other haramiyidans and *Megaconus*. A, Left P3 of *Arboroharamiya*. B, “Upper molariform tooth” of *Sineleutherus issedonicus* (PIN no. 5087/16) [15], which we think is most likely a right P3. C, Right M1 of *Arboroharamiya.* D, “Right upper molar” of *Eleutherodon oxfordensis* (M46832) [13], which we think is a left upper molar. E, Right m2 of *Arboroharamiya.* F, “Right lower molariform tooth” of *Sineleutherus issedonicus* (PIN, no. 5087/9) [15], which we think is a left lower m1 or m2. G, “Left lower molariform tooth” of *Sineleutherus uyguricus* (SGP 2001/33) [14], [31], which we think is a right m1 or m2. H, Left m3 of *Megaconus* (PMOL-AM00007) [28]. I, Upper dentition of *Megaconus* (PMOL-AM00007) [28]. The red arrow points to the mesial end of the upper molar. The blue arrow points to the distal end of the lower molar. The yellow points to the wear facets on the buccal (w1) and lingual (w2) wear facets of A1 (our interpretation). The cusp designation between brackets is our interpretation. Tooth images other than those of *Arboroharamiya* are from references cited. Images are not on the same scale.

Among haramiyidans *Arboroharamiya* is most similar to eleutherodontids (not including *Megaconus*). In our interpretation of the tooth orientation, they share the following derived characters: Lower molars are oval, with two rows of cusps that are continuous round the distal end. The largest lower cusp is at the mesial end of the lingual row (Figs. 13E, F, G). The sides of the occlusal grooves of upper and lower teeth, particularly the upper premolars, are covered with numerous minor transverse ridges (‘fluting’). Palinal occlusion was extensive, but an orthal component was retained.

*Arboroharamiya* differs from eleutherodontids in being larger and having more cusps on the lower molars, of which a1 is hypertrophied. The basin of lower molars is deeper and has stronger ridges derived from the cusps. The upper molar is wider mesially than distally and has only two rows of cusps that surround the mesial edge of the tooth. We consider that B1 of eleutherodontids should be A1 and tentatively homologize, by position, cusp BB of eleutherodontids with A5 of *Arboroharamiya* (Figs. 13C, D). Thus, the main differences of the upper molar between *Arboroharamiya* and eleutherodontids are that the former lacks a medial cusp row represented mesially by Bx and that the upper molar of *Arboroharamiya* has a single broad basin. Because of the cusp arrangement the upper molar outline of eleutherodontids is rhomboidal, whereas that of *Arboroharamiya* is roughly rectangular. These differences were sufficient in our view to establish a family Arboroharamiyidae [26].

### *Megaconus*

*Megaconus* [28] and *Arboroharamiya* [26] have been identified as haramiyidans, but studies of the two animals independently reached different conclusions on the phylogenetic positions of haramiyidans. In Zhou et al. [28] haramiyidans are separated from multituberculates and placed outside the Mammalia. In Zheng et al. [26], however, haramiyidans and multituberculates are clustered as Allotheria that was placed within the Mammalia, implying a Late Triassic origin of mammals.

Because these differences affect profoundly the phylogeny and our understanding of mammalian evolution [29], it is worthwhile comparing the two species in some detail. The comparisons focus on their dental and mandibular morphologies. The postcranial of *Arboroharamiya*, particularly the ankle region, is not well preserved, and the same region was preserved as impressions in *Megaconus* so that a comparison of the postcranial morphologies is not possible in this study.

*Arboroharamiya* differs from *Megaconus* in having only one lower premolar and two upper and lower molars, the basined upper cheek with the mesial end closed with small cusps, basined lower cheek teeth with distal end closed by small cusps, molars with conical rather than pyramidal cusps that have distinctively different sizes with A1 and a1 being hypertrophied, and enamel ridges (fluttings) on teeth, particularly on the upper incisor, premolars and upper molars. *Megaconus* is interpreted to have the postdentary trough and bones [28], which are absent in *Arboroharamiya.* Unlike *Megaconus* and some obligatory terrestrial mammals [28], the tibia and fibula of *Arboroharamiya* are not fused. Instead, the postcranial of *Arboroharamiya* is gracile, similar to that of *Haramiyavia* [25]. The manus and pes of *Arboroharamiya* are characterized by relatively short metapodials but long phalanges.

## Discussion

### Tooth Identification of Haramiyidans

Haramiyidans were previously known mainly from isolated teeth [10]–[15], [19], [20], [30], [32], [34], [38]. The discovery of *Haramiyavia* [25] helped to clarify identifications of isolated teeth whose assignments to various haramiyidan species were uncertain at the time [10], [11], [37], [39], [40]. However, the upper premolars of *Haramiyavia* were unknown and the differences between the upper premolar and molar of haramiyidans remained unclear.

As described above, the premolar differs from the molar in being more rounded with a broad central basin in *Arboroharamiya*. In addition, the premolar has fine ridges (flutings) on all sides of cusps, whereas the molar is roughly rectangular and has two rows of cusps that confine a mesiodistally elongate central basin in which there are transverse flutings. Moreover, the basin of the upper molar is closed mesially by cusps, as indicated by the red arrow in Figure 13, but open distally. The lower molar generally has an elongated oval shape in occlusal view and is closed distally by cusps (or U-ridge [19]), as indicated by the blue arrow in Figure 13. In light of the dentition of *Arboroharamiya*, it has been suggested [26] that some haramiyidan teeth identified as upper molariforms, such as those (specimens PIN 5087/16 and 5087/10) in Averianov et al. [15] (see also Fig. 13B) and the tooth (BDUC J 562) in Butler and Hooker [13], are probably premolars because they have the crown morphology similar to the upper premolars of *Arboroharamiya.* As mentioned above, the tooth that has been identified as an upper molar of *Eleutherodon oxfordensis* ([34]: Fig. 6) may be a lower molar, although the same tooth was considered as an M2 ([28]: Fig. S6).

### Tooth Orientation and Occlusion in Haramiyidans

In addition to the tooth morphologies and identification of the haramiyidan premolar and molar, there exists a major issue regarding the tooth orientation. Prior to the discovery of *Arboroharamiya* and *Megaconus*, *Haramiyavia* was the only taxon with teeth preserved in situ. In *Haramiyavia* a1 (the mesiolingual cusp) is the largest cusp on the lower molars, whereas the distobuccal cusps (A1) of upper molars are the largest ([25]: figs. 2, 4). *Thomasia* was considered to have a dental condition similar to *Haramiyavia* [19]. This condition is again present in *Arboroharamiya* [26]. However, in Eleutherodontidae, the hypothesized orientation of the isolated teeth is different.

**Figure 14:**
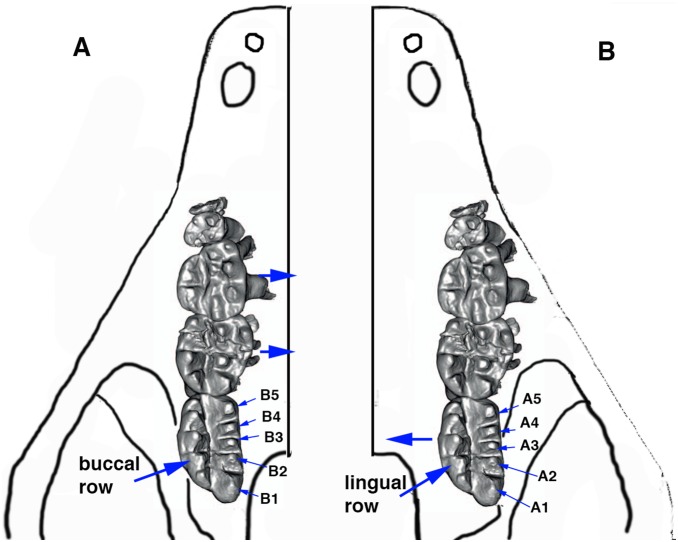
Orientations of the dentition in *Megaconus.* The hypothetic skull outline shows the palatal region where the occlusal views of the upper teeth are illustrated. In scenario A, which is the interpretation of Zhou et al. [28], the longer cusp row in M3 (row B) and the shortest cusp row in M2 are on the lingual side. Relative to the last molar, M1 and M2 shift lingually. In scenario B, which is our interpretation, the longer cusp row in M3 (row A) and the shortest cusp row in M2 are buccal. In relation to M1 and M2, M3 shifts lingually. The tooth images are from Zhou et al. [28]. A Jurassic mammaliaform and the earliest mammalian evolutionary adaptations. Nature 500: 163–167 (DOI: 10.1038/nature12429). Reproduced by permission of Nature Publishing Group.

The tooth orientation of *Eleutherodon* was first proposed by Kermack et al. [34], based on the upper tooth root condition and wear facets. In determining the upper tooth orientation, Kermack et al. ([34]: 585) wrote: “Among the teeth, EF FM/56 is unique in having long, well preserved roots with a strong lateral curve (Figs 1 and 14). In an upper molar the roots must follow the curve of the maxilla, i.e. curve medially. In no way could they curve laterally and remain within the bone. This makes EF FM/K56 a right upper molar and enables the rest of the teeth to be oriented, cusp A being distal and lingual.” These authors (34: Fig. 2) also noted “there is a true wear facet on what was deduced from the above to be the lingual side.” This wear facet is illustrated as 13 ([34]: Fig. 2A) and w1 in Figure 13C–D. In orienting the lower molar, Kermack et al. ([34]: 585) wrote: “In BDUC J. 461, a lower tooth, there is a facet (21a) on one of the lateral surfaces of the tooth: in all except highly specialized tetrapod dentitions, the upper teeth overhang the lower buccally; thus facet 21a would be on the buccal surface of the tooth and the β teeth now have been oriented, the largest cusp (cusp a) being mesial and buccal.” Butler [19] changed “cusp a” to cusp b2, because he thought that this cusp must have occluded in the basin between rows A and B of the upper tooth and was probably homologous with b2 of *Thomasia* on the buccal row. Similarly, Butler ([19]: 327) considered cusp A in the upper molar “could be homologized with B1 of *Haramiyavia* and *Thomasia.*” The tooth orientation of eleutherodontids has since been followed by others [13], [14], [28] (Figs. 13D, F, G), although the terminology, such as cusp BB and Bx, was used slightly differently in Butler and Hooker [13].

Kermack et al. ([34]: 586), however, noted that the tooth orientation of *Eleutherodon* differed from those of other haramiyidans and wrote: “This orientation contradicts that of the teeth of *Haramiyavia clemmenseni*, and probably that of haramiyidans, where the largest cusp is buccal on the upper molars and lingual on the lowers. However we consider that the morphological, chronological and geographical distances between the two samples may account for this discrepancy, and we estimate the above arguments as firmly established”.

Jenkins et al. [25] observed that a1 (mesiolingual) was the largest cusp on the lower molars of *Haramiyavia*, but still considered that the buccal row of the lower molar of *Haramiyavia* was used for occlusion. These authors [25] wrote: “From the distribution of wear facets and their relative wear, Hahn [7] correctly deduced (as did Parrington [5]) that the upper and lower teeth were offset to provide interlocking occlusion of rows and basins. Hahn also concluded from the relative wear on the facets that the lower of the two cusp rows on both upper and lower teeth (row B) made occlusal contact with the opposing basin. Although the teeth of *Haramiyavia clemmenseni* are only slightly worn, this occlusal pattern (Fig. 4) is verified as a feature common to haramiyidans and the earliest known true multituberculates.” The consensus view has therefore been that in haramiyidans row B (lingual) of the upper molar occludes in the valley between lower rows a and b of the lower molar, and in a similar manner row b (buccal) of the lower molar occludes between upper rows A and B [19].

Given the orientation of eleutherodontids, there are several questions that can be asked: Why was the largest cusp (a1) developed at the mesiolingual end of tooth but not used for occlusion in *Haramiyavia* and *Thomasia*? Why did extra cusps (C cusps) develop on the buccal side of row A in upper molars if only row B (lingual and lower) occlude with row b in *Haramiyavia*? On the other hand, why was the largest cusp developed on the buccal row (denoted as b2) and used for occlusion in eleutherodontids? Why is there a distinct wear facet on the lingual side of cusp A (sensu Kermack et al. [34]: Fig. 2), yet none on the lingual side of cusp B if the lingual row of upper cusps bites into the valley of the lower molar? What could “the morphological, chronological and geographical distances” [34] be, that would explain the difference between eleutherodontids and *Haramiyavia* plus other haramiyidans?

It has been difficult to orient isolated teeth of haramiyidans. The criteria used by Kermack et al. [34] and others were greatly restricted by the limited information from isolated specimens available at the time. We still do not know the morphology of the maxilla of eleutherodontids, even if *Megaconus* [28] is considered to be an eleutherodontid. Thus, it is still unknown how the long root of *Eleutherodon*, which appears to be a common feature for haramiyidans, was implanted in the skull. The root curvature at its distal end in EF FM/56, in our view, is not so significant; it is in the space of bone that contains a much thicker proximal portion of the root. The wear pattern of the lower molar of *Eleutherodon* on the buccal side of row b ([34]: Fig. 2B) is identical to that on the lingual side of row a in *Arboroharamiya* (Figs. 4, 11, 12). The wear pattern of the holotype upper molar of *Eleutherodon oxfordensis* ([34]: Fig. 2A) and those in Butler and Hooker ([13]; Fig. 13D) are also highly comparable to that of the right upper molar of *Arboroharamiya* (Figs. 9, 11, 12), except that the lingual and buccal sides were reversed. That upper molar was identified as the right one [13], but in our view it is a left upper molar. Originally, the wear facet (denoted as facet 13) on the lingual side of cusp A was identified ([34]: Fig. 2A). As observed by Kermack et al. [34] (p. 598) “The third wear facet, 13, lying on the lingual side of the upper tooth, begins at cusp A and becomes deeper as it passes mesially and eventually ends before the level of cusp B.” This wear pattern is identical to that of the upper molar of *Arboroharamiya* if the left tooth was considered as the right, or vice versa. The wear on the buccal side of A in *Eleutherodon* was not illustrated. This is because Kermack et al. [34] emphasized the lingual wear in order to determine the orientation of the tooth. Nonetheless, it was noted that a wear facet in the larger tooth valley (facet 11) cuts through the distal margin of the tooth [34], which is on the buccal side of cusp A. The buccal wear of cusp A is clearly shown in the SEM photographs ([13]: Fig. 1A, B) and is illustrated as w2 in Figure 13C and D. The wear facets on both sides of the large distal upper cusp (denoted as B1) of *Eleutherodon* were also illustrated ([19]: Fig. 5B). The wear facets denoted as w1 and w2 (Figs. 13C, D) on the lingual and buccal side of the main distal cusp are unique, and can be created only if this cusp bites into and “moves” along the valley of the lower molar in a fashion illustrated in Figure 11.

In light of the evidence from *Arboroharamiya*, it is clear that the tooth of previously known eleutherodontids has been incorrectly oriented. What has been identified as a left molar is actually a right one, and vice versa. In other words, “b2” of the lower molar in known eleutherodontids should be a1, and B cusps in known eleutherodontids should be A cusps, as we indicate in Figure 13 (D, F, G). With the correction, the cusp patterns that a1 is the largest cusp of lower molars and that the largest cusp is distobuccal on upper molars become consistent within haramiyidans. With the new orientation, the occlusal wear and masticatory patterns of *Eleutherodon* ([34]: Fig. 23) become highly similar, if not identical, to those of *Arboroharamiya* ([26]), as shown in Fig. 11.

Late Triassic *Haramiyavia* and *Thomasia* are more primitive in that they have or were interpreted to have more teeth in their dentitions. For instance, *Haramiyavia* has a lower dental formula of i4-c1-p4-m3 [25]. In addition, the upper molars of *Haramiyavia* have extra cusps (cusp C) developed on the buccal side of cusp row A. Although *Arboroharamiya* and other new haramiyidans [41] are more derived in both tooth and mandibular morphologies than *Haramiyavia,* we think the new data allow us to entertain alternative interpretations for the occlusal pattern in *Haramiyavia* and tooth identification and orientation in *Thomasia*.

For *Haramiyavia*, contrary to the interpretation of Jenkins et al. [25], it is highly probable that the lingual cusp row of the lower molar occluded with the valley between cusp row A and B of the upper molar. There are several lines of evidence to support this interpretation. First, this reinterpretation is consistent with the occlusal pattern of *Arboroharamiya* [26] (this study) and other new haramiyidans in which the teeth are preserved in situ [41]. It is more parsimonious to assume that the occlusal pattern did not change during the evolution of haramiyidans. In other words, *Arboroharamiya* retained the occlusal pattern of *Haramiyavia*, although the former became more specialized with a hypertrophied cusp a on lower molars. Secondly, if the lingual (B) cusp row of the upper molars occludes with the buccal row of the lower molar, as interpreted by Jenkins et al. [25], it would be difficult to explain why cusp a1 in the lower molar is the largest and why extra C cusps developed on the buccal side of row A in upper molars of *Haramiyavia*. A large cusp a1 unused in occlusion, and the addition of cusps on the non-functional buccal side of the upper molar, make little functional sense. Finally, it is generally true that in multituberculates the primary functional cusp row usually bears the largest cusps, particularly so for the upper molars. For M1 teeth with only two upper cusp rows in multituberculates the lingual row usually has the more robust cusps. For M1 teeth with a secondary lingual ridge or cusp row, as in many cimolodontan multituberculates, the medial row (the original lingual row) still retains the strongest cusps. The occlusal condition of the M2 of multituberculates is different. The buccal cusp row of M2 is the primary functional row and it bears the largest cusps regardless of whether there is a secondary ridge or cusp row developed on the buccal side of the original buccal cusp row. Compared with the multituberculate dentition, it is most likely that cusp row A in the upper molar of *Haramiyavia* is the primary functional row because it bears the strongest cusps. It is highly probable that row A of *Haramiyavia* occluded with the valley of the lower molar, which also means that row a of the lower molar occluded with the valley between row A and B of the upper molar. In this occlusion, the buccal (b) cusp row of the lower molar would occlude with the valley between row A and C cusps of the upper molar, which explains why C cusps are developed on the buccal side in upper molars of *Haramiyavia.* Phylogenetically and functionally this interpretation is meaningful.

Comparison with teeth of *Thomasia* is more difficult because the genus was based exclusively on isolated teeth. Identification and orientation of those teeth were affected by the conventional view that the buccal cusp row of the lower molar occluded with the lingual cusp row of the upper molar (e. g. [25]). In light of the new data from *Arboroharamiya* [26] and other euharamiyidans [41] we consider some teeth previously identified as upper molars of *Thomasia*, such as M214 and M2401C [11], to be most likely right lower molars. This is because the general morphology of these teeth is comparable to the lower molars of *Arboroharamiya* in which the mesiolingual cusp (cusp a1 in our interpretation) is inflated as the largest tooth cusp and is positioned near the longitudinal axis of the tooth. The wear facets indicated by arrows on those specimens ([11]: figs. 3–5) would be reinterpreted as on the lingual side of the lingual cusps, similar to those of *Arboroharamiya*. We consider some other specimens, such as M216, M222, SNP59, SNP350 and SNP660 in Butler & Macintyre [11], to be most likely upper molars because these teeth have the largest cusps (A1 and B1 in our interpretation) more distantly separated at the distal end of the tooth so that the valley of those teeth is open distally to allow palinal movement of the lower tooth, again as in *Arboroharamiya*. Our reinterpretation of the teeth of *Thomasia* implies that the two sets of teeth originally assigned to *Thomasia* and “*Haramiya”,* respectively, may not be exclusively from opposing dentitions of the same genus [10]; instead, each set of the two may contain both upper and lower teeth of the same genus or even different genera. The teeth currently assigned to *Thomasia* show a considerable variation; it is perhaps necessary to reexamine and identify those teeth in light of the tooth morphology and orientation of *Arboroharamiya* and other new evidence. Reexamination may result in reinterpretation of those teeth and thus cast new light on the evolution of the allotherian tooth pattern, which remains one of the most difficult problems existing in the study of mammalian evolution.

### Taxonomy of *Megaconus*

Although *Megaconus* has fragments and impressions of skull and skeletal elements preserved in split slabs, its identification as an eleutherodontid haramiyidan was mainly based on dental morphologies. This is because haramiyidan taxa are principally based on isolated teeth and diagnosed primarily by dental characters. Zhou et al. ([28]: Supplementary Information) wrote: “Among mammaliaforms *Megaconus* is most similar to *Thomasia*, *Haramiyavia* and *Theroteinus*, but differs from these forms in having three functional cusp rows on upper molars 1 and 2 instead of two rows on upper molars as in *Thomasia*, and two functional rows plus labial cuspules with occlusion as in *Haramiyavia* and *Therotenius*. Most similar to *Eleutherodon* and *Sineleutherus* in similarity of hypsodont roots of molars, and multiple roots that are proximally confluent, and in having an enlarged anterior cusp on the labial row, …”.

We can list more differences between *Megaconus* and other haramiyidans, but our discussion will focus on Eleutherodontidae, in which *Megaconus* was placed. After considering the original diagnosis for Eleutherodontidae [19], which we quoted above, we found that previously known eleutherodontids and *Megaconus* share only one character “in having an enlarged anterior cusp on the labial row” of lower molars. Because the lower tooth with occlusal view preserved (as a CT-image) is the left m3 (Fig. 13H) whose mesial end was broken, the sizes of the mesial cusps are not certain. Judging from the trench-like straight groove on M3 in which the lower cusps would fit into, it can be estimated that the mesial cups of m3 in *Megaconus* must not be greatly enlarged. In all eleutherodontids previously known, the mesial cusp of the lower molar is enlarged because a broad basin in the upper molar can accept it.

The tooth orientation of *Megaconus* can be determined by the tooth position in the dentary. If the mesiobuccal cusp in *Megaconus* is indeed enlarged, then this enlarged cusp is different from the condition in previously known eleutherodontids. As we demonstrated in the previous study [26] and here (see above), the enlarged cusp on the lower molar of previously known eleutherodontids should be a1 (mesiolingual), not b2. Thus, the enlarged mesiobuccal cusp in the lower molar of *Megaconus* actually differentiates it from other eleutherodontids.

The other feature that Zhou et al. [28] used to group *Megaconus* with eleutherodontids is the “hypsodont roots of molars and multiple roots that are proximally confluent.” The root condition was not previously considered one of the diagnostic features for Eleutherodontidae [19]. Among haramiyidans where the tooth roots are known, a similar root condition exists in *Eleutherodon* [13], [34], *Sineleutherus uyguricus* [14] and in *Arboroharamiya*, as we show in this study. To cite one more example, the m2 of *Hahnodon taqueti* [42] has a root condition similar to that of *Megaconus* ([28]: Fig. S4J). *Hahnodon taqueti* was originally placed in the family Hahnodontidae [42], which also contains *Denisodon moroccensis* [43], and the family was considered a member of the paulchoffatioid multituberculates [4]. Butler and Hooker [13] removed Hahnodontidae from Multituberculata to Haramiyida because hahnodontids have “basined wear” on their teeth. However, Hahn and Hahn [20] disagreed and retained Hahnodontidae within multituberculates because their tooth structures, such as “two large anterior cusps closely attached to each other”, differentiate them from haramiyidans. Unfortunately, other tooth roots of *Hahnodon taqueti* are unknown, but its m2 root condition lends support to Butler and Hooker’s [13] view if the proximally fused roots represent a haramiyidan condition. If so, then the root condition is not exclusive to *Megaconus* and previously known eleutherodontids but is found more widely across derived haramiyidans.

The last feature that links *Megaconus* with eleutherodontids is its interlocking mechanism between adjacent molars [28]. In *Megaconus* the upper teeth, particularly between M1 and M2, have a clear interlocking relationship, but that relationship is not so obvious in eleutherodontids ([28]: Fig. S6D). Because eleutherodontids are known only from isolated teeth, whether they actually have the interlocking feature is subject to future testing. The tooth identified as an upper molar of *Eleutherodon oxfordensis* ([34]: Fig. 6, BDUC J.185) was regarded as an M2 ([28]: Fig. S6D), but that tooth in our view could be a lower molar because its fusiform outline differs from the rhomboidal shape of the upper molar.

While few similarities were present to support the taxonomic position of *Megaconus* as a eleutherodontid haramiyidan, Zhou et al. [28] listed several differences between them: *Megaconus* “differs from *Eleutherodon* and *Sineleutherus* in having pyramidal cusps of subequal size, and straight valleys, instead of fusiform valleys or basins and the large and anteriorly curved last cusp on the upper molars, and a single recurved and enlarged anterior cusp and coalesced marginal cusps on the lower molars.” It is also noted that *Megaconus* is different from other eleutherodontids in lacking the dense flutings on the tooth crown ([28]: 163), although enamel flutings are variable in presence within eleutherodontids [14], [21]. Moreover, if our interpretation of the upper premolars in other eleutherodontids [13] is correct (Fig. 13A–B; see below), then eleutherodontids are distinct from *Megaconus* in having basined upper premolars with enamel flutings. Our conclusion is that the placement of *Megaconus* in the family Eleutherodontidae needs to be tested by future phylogenetic analyses.

While *Megaconus* is similar to multituberculates in having pyramidal cusps of subequal size and straight valleys between cusp rows, several characters have been cited to distinguish *Megaconus* from multituberculates [28]. Of those features, there are two critical ones from the molar morphologies: *Megaconus* was interpreted to have three molars and M1-2 are lingually offset from M3. In contrast, there are only two upper and lower molars in multituberculates and the most important synapomorphy of all multituberculates is that M2 is offset lingually to M1. However, we found that these differences are highly questionable, as discussed below.

### Tooth Orientation of *Megaconus*

In the original publication, Zhou et al. [28]: wrote: “fully preserved P2-M3 from the left side of skull in lingual view and exposed in situ after initial preparation.” In the separate caption associated with figure S2, it is also noted as: “Skull and dentition (before excavation of left upper P1-M3, and left lower m1–m2).” The left upper dentition was confirmed by one of us (JM), who examined the holotype specimen. In a later corrigendum (doi:10.1038/nature12812) by Zhou et al., however, several changes were made, of which the most important one is that the upper dentition in question was re-described as from the right side of the skull, instead of from the left side of the skull. These changes have been inserted in the online version of the paper [28]. However, after the publication of the corrigendum, one of us (YW) examined the holotype specimen again and came to the same conclusion that the upper dentition in question is the left one.

Using the animal’s anatomical posture as the reference, the main part of the split holotype slab of *Megaconus* (PMOL-AM00007A) would be considered as the left slab, and the counterpart (PMOL-AM00007B) is the right one. Under the new interpretation of Zhou et al. [28], the left slab contains the right skull with the right upper dentition, partial left upper dentition and the left mandible with partial left lower dentition as well as with its exposed medial surface. The right slab contains the left skull with partial impression of the right upper dentition, most of the left upper dentition and the mandible (presumably the right one) with its lateral surface exposed (as indicated by the masseteric fossa). In the revised on-line paper [28], however, inconsistencies still exist in several places. For instance, in the Supplementary Information containing figures, the caption attached to figure S2 indicates that the image of P2-M3 in S2A is the lingual view, contrasting the image in S2D and to the orientation of the same dentition in S2C. In S2C, the lingual side shown in S2A is indicated as the labial side. Another example is that in the caption of figure S6 of the revised paper [28], the image of P2-M3 is still noted as “reversed from the left teeth on PMOL-AM00007A”, whereas the “reversed” P2-M3 in figure S6A–C is shown as a left dentition.

Given our observation and the original study [28], there are thus two interpretations about the orientation of the upper dentition in *Megaconus*: one by Zhou et al. [28] (Fig. 14A) and the other by us (Fig. 14B). The difference of the two interpretations affects fundamentally the morphology of *Megaconus* and thus its taxonomic and phylogenetic placement. Further investigation of the specimen and discovery of new specimens are needed to resolve this discrepancy.

### Toothrow and jaw length of *Megaconus*

It was reported that *Megaconus* has two premolars and three molars in the lower and upper jaws [28]. Under their interpretation the ultimate lower premolar is much smaller than the mesial (penultimate) one, a condition unknown in any early mammals or mammaliaforms. Such a lower premolar condition is also inconsistent with the upper dentition in which there are two small premolars. Zhou et al. [28] wrote: “*Megaconus* is named after the unique and enlarged anterior cusp on the first lower premolar for shearing and puncturing, morphologically distinctive from multicusp rows on the lower molars.” However, in their figure 2d and e [28], it is difficult to imagine which upper tooth the enlarged p1 can occlude with. Furthermore, the small length mismatch between the upper and lower postcanine toothrows, according to the dental interpretation of Zhou et al. [28] and shown in their figures 2D and 2E, is a considerable underestimation of the actual length mismatch under such an interpretation. In the original study of *Megaconus* [28], tooth measurements were not provided. Nonetheless, the postcanine toothrow length can be estimated from figure S2D [28] using the interpreted dental formula and the scale associated with the image. The lower postcanine toothrow length is estimated as 18 mm, whereas that of the upper is 12.7 mm. The upper toothrow length is about 70% of the lower. In particular, the estimated m1 length (5.8 mm) is more than twice of the estimated M1 length (2.7 mm). This large length mismatch between upper and lower toothrows further aggravates the problem of the occlusal relationships. Figure S2D is an outline tracing from figure S2E [28], a photograph of the actual specimen in the same orientation, so measurements obtained from S2D should be relatively accurate. The large difference in length between upper and lower toothrows was however scaled down for dental reconstructions in figures of the primary (non-supplementary) publication, without explanation ([28]: figs. 2d–e, 4).

Associated with the long lower dentition, the dentary bone as interpreted in the original study [28] is also unnaturally long, compared to the skull length ([28]: figs. S2, S3). If the mandibular condyle were fitted to the glenoid fossa, roughly at the position of the squamosal zygoma, the lower premolar would occlude with the upper incisors and the lower incisor would certainly be located much anterior to the upper incisors.

Having examined the specimen, we think that these differences cannot be attributed to differential distortion of the skull and dentary. Instead, it is likely that these unusual conditions stem from mis-interpretations of the dentary bones and lower dentition of *Megaconus.* Direct comparison of the specimen photograph in figure S2E [28] and the interpreted line drawing (S2D) with the specimen shows that it is highly probable that the reconstruction is composed of parts from both dentary bones. The actual shape of the dentary is more likely the one outlined (in white line) in figure S3B [28], if the “right” i1 ([28]: S3C) is interpreted as the left one.

As currently interpreted [28], the long lower dentition and dentary make little sense when mastication is concerned. The tooth row of *Megaconus* shows horizontal longitudinal wear grooves, which implies palinal molar occlusal movement. As a character used in the phylogenetic analysis, the direction of the lower jaw movement during occlusion was coded as dorsoposterior for *Megaconus*, similar to multituberculates [28]. It is difficult to interpret how the lower jaw functions if the lower dentition is so much longer than the upper one and the mandible is at least as long as the skull.

### Number of Molars in *Megaconus*

We consider that the identification of the upper dentition as having three molars in *Megaconus* is not conclusive. *Haramiyavia* has three upper and lower molariform teeth [25] and by analogy with *Haramiyavia*, *Thomasia* was considered to possess three molariforms [19]. The number of molars is uncertain in other haramiyidans [20]. *Arboroharamiya* has only two lower and upper molars, which is similar to multituberculates but differs from *Haramiyavia*.

In *Megaconus*, the tooth identified as M1 may be the ultimate premolar for at least two reasons. First, the cusp shape between M1 and M2-3 is different. M1 cusps are somewhat conical, whereas those of M2-3 are pyramidal with subequal size (Figs. 13I, 14). It was also noted that the lower molar cusps are mostly of pyramidal shape [28]. Second, the wear pattern of these teeth is considerably different. As noted by Zhou et al. ([28]: Supplementary Information): “The first upper molar shows considerable apical wears on most cusps. M2 and M3 show extensively developed wear facets on flanks of cusps in each cusp row and these facets are aligned on longitudinal furrows.” The differences in cusp shapes and wear patterns indicate that M1 and M2-3 function differently in chewing. Because of these differences, the possibility that *Megaconus* has only two upper and lower molars should not be ruled out.

In addition, the M2-3 cusp shape, size, arrangement and wear pattern are significantly different from the basined molars of eleutherodontid haramiyidans but more similar to those of multituberculates [4]. If the basined wear can be used as a character to move Hahnodontidae from the Multituberculata to the Haramiyida, as suggested by Butler and Hooker [13], then the tooth morphologies of *Megaconus* strongly suggest affinity with multituberculates.

Furthermore, the condition that M1-2 lingually offsets M3 was used to distinguish *Megaconus* from multituberculates [28] under the interpretation of Fig. 14A. If instead the interpretation we suggest in Fig. 14B is correct, then it is M3 that offsets lingually to M1-2, which is “the most important synapomorphy of all multituberculates” [28].

### Mandible and Middle Ear of Haramiyidans

A more detailed image (Fig. 2C) reemphasizes our confidence that the dentary of *Arboroharamiya* lacks the postdentary trough or the groove for the Meckel’s cartilage [26]. Previously, the only known haramiyidan dentary was that of *Haramiyavia* [25], which was considered to resemble that of *Morganucodon* and *Kuehneotherium* in several aspects: the masseteric fossa does not extend beyond the posterior part of the last molar, the condyle is above the level of the teeth, and most importantly the postdentary-trough is present [19], [20]. The latter is one of the characters differentiating haramiyidans from multituberculates [25]. The dentary of *Arboroharamiya* is highly specialized and drastically different from that of *Haramiyavia,* but resembles those of derived multituberculates such as taeniolabids [4] in being short and deep and having only one lower premolar. Most importantly, it does not have the postdentary-trough. Lack of the postdentary trough suggests formation of the definitive mammalian middle ear (DMME [44], [45]). If *Haramiyavia* is a basal haramiyidan whereas *Arboroharamiya* represents a derived species, then the similarities of the lower jaw and formation of the DMME between *Arboroharamiya* and those of multituberculates and other mammals display a mosaic evolutionary pattern. Under this phylogenetic hypothesis the DMME in allotherians must have evolved independently from that of therians and monotremes. This is not a surprise since independent evolution of the DMME has been implied at least in Yinotheria within the crown mammals [46].

Whether *Haramiyavia* has the postdentary trough is a matter of debate. Averianov et al. ([15]: 105–106) argued that “However, a groove of *Haramiyavia* that was proposed to contain postdentary bones [10; Fig. 2] is not suitable for this, because it is located anteriorly rather than posteriorly to the mandibular foramen. This groove likely contained Meckel’s cartilage. In *Haramiyavia*, the bone lacks a fragment posterior to the mandibular foramen; thus, it is impossible to recognize whether or not it had a groove for postdentary bones.” In discussing the transitional mammalian middle ear (TMME) in eutriconodonts, Meng et al. [47] have pointed out that most of the prearticular of *Morganucodon,* as interpreted by Kermack et al. [48], [49], is probably the ossified Meckel’s cartilage, which is located distoventral to the mandibular foramen ([47]: Fig. 2a). This notation gains support from Zhou et al.’s ([28]: Fig. S3) reconstructions of the lower jaws of *Sinoconodon* and *Morganucodon*, in which the postdentary bones are located posterior to the mandibular foramen and the Meckel’s cartilage is located anteroventral to the ultimate molar. Given the interpreted location of Meckel’s cartilage in *Sinoconodon* and *Morganucodon*, it is highly possible that the groove on the medial side of the fragmentary dentary of *Haramiyavia* ([25]: Fig. 2) is for the ossified Meckel’s cartilage (OMC), as suggested by Averianov et al. [15], even though the groove appears deeper than those that have the OMC actually preserved, such as *Repenomamus* [50], [51], *Yanoconodon* [46] and *Liaoconodon* [47]. Because only a short segment of the dentary containing m3 is preserved in *Haramiyavia*, we cannot rule out the possibility that the groove in *Haramiyavia* accommodated the OMC, not the postdentary bones.

The postdentary trough and bones were interpreted to be present in *Megaconus*. Although we are not convinced after examination of the specimen by two of us (JM, YW), we choose not to reinterpret those uncertain structures but wait for new material to prove the contrary. We do however raise the concern that, if the postdentary bones do exist in *Megaconus* as interpreted [28], there is a need to explain how the lower jaw function with the mandibular ear attached.

*Morganucodon* was known to have a “double” jaw joint: the dentary-squamosal (secondary) articulation on the lateral side and the articular-quadrate (primary) articulation on the medial side [48], [49], [52]. A similar double joint probably exists in docodontans [53], [54], and the yinotherian *Pseudotribos* [55]. Even in eutriconodonts, such as *Liaoconodon* [47], the primary joint may also have been affected by jaw mastication. In all these forms, however, the secondary craniomandibular joint had a hinge-like movement when the jaw opened up, which can be inferred from tooth morphology and the shapes of the mandibular condyle and glenoid fossa. In contrast, the mandibular condyle of *Megaconus* is ventrodorsally deep. Zhou et al. ([28], Suppl. Info.) stated that “the craniomandibular joint is formed by the dentary condyle and the squamosal glenoid, in addition to the quadrate (incus) and the articular (malleus) joint.” As inferred from tooth morphology and wear facets, Zhou et al. [28] also concluded that *Megaconus* has dorsoposterior jaw movement, as a derived character shared with multituberculates. This raises a challenging question on the mechanism of jaw movement and hearing in *Megaconus.*

## Conclusions

We present a detailed description of the mandible and dental morphologies of *Arboroharamiya jenkinsi,* an effort to follow up the recent study that established the species [26]. This is particularly pivotal because the study of Zheng et al. [26] and that of Zhou et al. [28] independently reached conclusions that differ significantly about the phylogeny and divergence time of the Mammalia. The two conclusions cannot be both right and are subject to future testing.

The evidence shows that *Arboroharamiya* is indeed a specialized haramiyidan. Its morphology casts new light on various existing problems in the study of haramiyidans and early mammals. At the same time, we explored various morphological features of *Megaconus.* We suggest that aspects of its dental morphology were unclear or erroneous in the original study [28] and that alternative interpretations and hypotheses regarding its morphology should be considered. We also suggest that the possibility of *Megaconus* as a multituberculate should not be ruled out and that the postdentary bones in *Megaconus*, if indeed present, require a functional explanation. A more conclusive view on all of these issues would benefit from additional material and evidence.
